# Differential expression profile of genes involved in the immune response associated to progression of chronic Chagas disease

**DOI:** 10.1371/journal.pntd.0011474

**Published:** 2023-07-13

**Authors:** Inmaculada Gómez, Manuel Carlos López, Adriana Egui, Génesis Palacios, Bartolomé Carrilero, Celia Benítez, Marina Simón, Manuel Segovia, Emma Carmelo, M Carmen Thomas

**Affiliations:** 1 Instituto de Parasitología y Biomedicina López-Neyra, CSIC, Granada, Spain; 2 Instituto Universitario de Enfermedades Tropicales y Salud Pública de Canarias, Universidad de La Laguna, La Laguna, Spain; 3 Unidad Regional de Medicina Tropical, Hospital Universitario Virgen de la Arrixaca, Murcia, Spain; 4 Departamento de Obstetricia y Ginecología, Pediatría, Medicina Preventiva y Salud Pública, Toxicología, Medicina Legal y Forense y Parasitología, Universidad de La Laguna, La Laguna, Spain; Universidade Federal de Minas Gerais, BRAZIL

## Abstract

**Background:**

Patients with chronic Chagas disease present marked clinical and immunological heterogeneity. During the disease, multiple immune mechanisms are activated to fight the parasite. The purpose of this study was to investigate the expression patterns of genes involved in relevant immunological processes throughout the disease in patients with chronic Chagas disease.

**Methodology/Principal findings:**

High-throughput RT-qPCR with QuantStudio 12K Flex real-time PCR system was used to evaluate the expression of 106 immune-related genes in PBMC from a cohort of cardiac Chagas disease patients (CCC I), asymptomatic patients (IND) and healthy donors (HD) after being stimulated with *T*. *cruzi* soluble antigens. Principal component analysis (PCA), cluster analysis and volcano plots were used to identify differentially expressed genes. In addition, gene set enrichment analysis (GSEA) was employed to identify the enriched immunological pathways in which these genes are involved. PCA revealed the existence of a statistically divergent expression profile of the 36 genes correlated with PC1 between CCC I patients and HD (*p* < 0.0001). Differential gene expression analysis revealed upregulation of 41 genes (expression fold-change > 1.5) and downregulation of 14 genes (expression fold-change < 0.66) (*p* = 8.4x10^-13^ to *p* = 0.007) in CCC I patients *versus* HD. Furthermore, significant differences in the expression level of specific genes have been identified between CCC I and IND patients (8 up and 1 downregulated). GSEA showed that several upregulated genes in CCC I patients participate in immunological pathways such as antigen-dependent B cell activation, stress induction of HSP regulation, NO2-dependent IL12 pathway in NK cells, cytokines-inflammatory response and IL-10 anti-inflammatory signaling.

**Conclusions:**

Cardiac Chagas disease patients show an antigen-specific differential gene expression profile in which several relevant immunological pathways seem to be activated. Assessment of gene expression profiles reveal unique insights into the immune response that occurs along chronic Chagas disease.

## Author summary

Among the neglected tropical diseases, Chagas disease is a major public health problem. The infection, caused by the parasite *Trypanosoma cruzi*, affects about 6–7 million people. The disease courses with an initial acute phase which, in the absence of treatment, becomes chronic. The chronic phase is initially asymptomatic, but in 40% of patients the disease progresses to clinical manifestations, being the most frequent and severe those affecting the cardiac system. The mechanisms underlying the development of cardiac disease are not yet fully understood, but this could depend on an unbalanced infection-triggered immune response. In this study, we have determined the expression level of 106 immune-related genes in patients in an early stage of cardiac disease (CCC I) and compared them with the expression present in asymptomatic patients (IND) as well as in healthy donors (HD). The results obtained have allowed us to identify a differential gene expression profile in chronic patients, as well as the identification of relevant immune pathways in which these genes participate. Therefore, our results contribute to the knowledge of the immunological mechanisms that are activated along chronic Chagas disease and, as a consequence, will help in the clinic management of the patients affected by this disease.

## Introduction

Chagas disease is a neglected tropical infection caused by the parasitic protozoan *Trypanosoma cruzi* that affects about 6–7 million people worldwide and with approximately 75 million people at risk of infection [1]. It is endemic in 21 Latin American countries and is considered an emerging infection as cases have been reported outside the endemic areas of transmission, primarily in the United States and Europe, as a result of migration and globalization processes [1–5]. The infective forms of the parasite are eliminated in the urine and feces of its transmitting vector during the bite and blood meal, and introduced into the host blood stream. After infection, Chagas disease patients enter into a 4- to 8-week acute phase, characterized by mild non-specific symptoms and a high parasitemia, which induces a strong activation of the immune system, including high levels of plasmatic cytokines, intense activation of B and T lymphocytes, and inflammatory reactions in the infected tissues [1]. The immune response fails to complete *T*. *cruzi* clearance, leading if untreated, to the asymptomatic chronic phase [6] which comprises a period of between 10 to 25 years. In this phase, exists a fragile balance between the host immune response and parasite replication that keeps patients in a clinically silent asymptomatic stage. The loss of this balance in 30–40% of patients is crucial for the progression of the sickness leading to the development of clinical manifestations with alterations involving the cardiac, digestive and/or nervous system [7]. The cardiac form, also known as chronic Chagas cardiomyopathy (CCC), is the most frequent and severe form of the symptomatic disease, being a great cause of morbidity and mortality, with a considerable socio-economic burden [7–9]. Following a criterion based on electrocardiogram (ECG) abnormalities, thorax X-ray and the presence of symptoms, patients with cardiac involvement can be stratified into 4 stages [10]. Group 0 (G0) includes chronic patients with a normal ECG and a chest X-ray, group 1 (G1) includes those with normal chest X-ray but abnormalities in the ECG, group 2 (G2) contains patients with ECG abnormalities and heart enlargement as determined by chest X-ray and group 3 (G3) to patients exhibiting ECG abnormalities, heart enlargement and clinical or radiological evidence of heart failure.

The mechanisms underlying the development of CCC are not yet fully understood, but its onset may depend on unbalanced *T*. *cruzi* triggered immune response and disruption of intrinsic heart factors [11]. Growing evidence suggests that parasite persistence is implicated in the establishment of the CCC form, as well as in its severity, by driving the immune response with production of several molecules that results in the activation of inflammatory mechanisms that are certainly involved in tissue damage, remodeling and fibrosis, resulting in progressive heart rhythm disturbances, ventricular dysfunction, aneurysms and thromboembolism [12]. Along the chronic phase, immune system cells trigger a cascade of events to control the parasite and restore homeostasis. To this end, Th1/proinflammatory cytokines are produced, both in infected mice and in Chagas disease patients [12]. Thus, elevated plasma levels of TNFα and peripheral blood mononuclear cell-derived IFNγ are identified in CCC and IND patients [13–15], most likely as a response to parasite persistence. In addition, it has been reported that this Th1-type immune response is particularly strong in patients with chagasic cardiomyopathy compared to IND patients [12]. On the other hand, it has been described that a regulatory response, via IL-17, may be relevant in the control of cardiac inflammation by playing a negative feedback role on the production of TNFα, IFNγ and chemokines during experimental *T*. *cruzi* infection, modulating the cardioimmune pathology of Chagas disease [16]. In addition, together to the inflammation processes in the cardiac tissue, the virulence and tropism of the *T*. *cruzi* infecting strain have been proposed as the main factors for the establishment and severity of the cardiomyopathy.

The identification of reliable markers for the prognosis of chronic Chagas cardiomyopathy development is one of the most important challenges in Chagas disease, driving the search for “immunological biomarkers” to predict the progression of CCC, allowing better clinical management and pharmacological intervention of patients [17,18]. In addition, with advances in genomics, “genetic biomarkers” have also been investigated. However, although many target molecules have been studied, few biomarkers of CCC have been currently proposed. The response of the host immune system to *T*. *cruzi* invasion may be the most important factor determining the severity of chronic disease [11], thus, the excessive or decreased production of mediators, such as cytokines, chemokines, adhesion molecules, and cellular activation markers, among others, may lead to a potential worsening or stabilization of CCC [18].

Since molecular mechanisms involved in the immune response play an important role in chronic Chagas disease, in this study we aimed to identify gene expression patterns that are involved in chronic Chagas disease patients with the early cardiac form (Kuschnir I, CCC I). The methodology used is based on high-throughput real-time qPCR to determine the expression level of 106 immune system-related genes in human peripheral blood mononuclear cells (PBMCs) samples from patients with CCC I and compared to that from healthy donors (HD), and subjects with indeterminate form of Chagas disease (IND). These studies have allowed us to identify antigen-specific differential gene expression patterns in chronic CCC I *versus* IND patients and *versus* HD involving a broad set of immune system-related genes that are members of several relevant immunological pathways. Studying the expression changes these genes undergo and the immunological pathways in which they are involved will improve our understanding of the immune mechanisms activated along chronic Chagas disease, providing novel insights into the pathogenesis of this neglected disease, as well as representing potential biomarkers of disease progression.

## Material and methods

### Ethical considerations

The protocols used in this study were approved by the Ethics Committees of the Consejo Superior de Investigaciones Científicas (Spain—Reference: 013/2020) and of the Hospital Virgen de la Arrixaca (Murcia, Spain—Reference: 2020-1-11-HCUVA). A signed informed consent was obtained from all voluntary patients and healthy donors before their inclusion in the study.

### Study cohort

The adult chronic Chagas disease patients originally from endemic areas and residents of Spain included in this study were recruited, diagnosed and clinically evaluated in the Hospital Virgen de la Arrixaca (Murcia, Spain). These patients, who had not received antiparasitic treatment, were diagnosed out according to the WHO criteria based on two conventional serological tests (Chagas ELISA, Ortho Clinical Diagnosis, and Inmunofluor Chagas, Biocientífica, Argentina) and stratified according to the Kuschnir grading system [10], being characterized as cardiac (CCC I) patients (G1 according to the Kuschnir classification) or indeterminate (IND) patients due to the absence of cardiac or digestive manifestations (G0 according to the Kuschnir classification). Also, healthy donors from endemic and non-endemic areas were included in this study. Table 1 shows the data referring to the age, sex distribution and country of origin of each of the subjects enrolled in this study.

**Table 1:** Epidemiological and demographic data of the study cohort.

Patients group		Origin (number; %)	Age (years)		Sex [% Female (F) / Male (M)]
			**Mean (± SD)** **Median (IQR)**	**Range**	
**Healthy donors** (n = 34)	**From non-endemic area** (n = 20)	Spain (20; 100%)	37.6 (12.8) 37.5 (23)	22–56	60% F
					40% M
	**From endemic area** (n = 14)	Colombia (4; 28.6%)	37.5 (8.4) 38 (8)	21–54	64.3% F
		Venezuela (2; 14.3%)			
		Chile (1; 7.1%)			
		Panama (1; 7.1%)			35.7% M
		Ecuador (1; 7.1%)			
		ND (5; 35.7%)			
**Indeterminate patients** (n = 71)		Bolivia (66; 93%)	34.8 (8.5) 34 (11)	18–59	70.4% F
		El Salvador (1; 1.4%)			
		Paraguay (1; 1.4%)			29.6% M
		ND (3; 4.2%)			
**Cardiac patients (K1)** (n = 32)		Bolivia (23; 71.9%)	36.4 (9.4) 32 (13.3)	21–55	59.4% F
		Ecuador (1; 3.1%)			
		Paraguay (1; 3.1%)			40.6% M
		ND (7; 21.9%)			

ND No data

A total of 18 samples from 32 chronic cardiac Chagas disease patients, 39 samples from 71 chronic indeterminate patients and 30 samples from 34 healthy donors [15] were included. Due to the quantity of RNA required to carry out the cDNA synthesis for high-throughput RT-qPCR and the limited number of cells isolated from the small volume blood samples of particular patients, in some cases, it was necessary to blend cells from some patients. The collection of new blood samples was not possible in any case since the patients with Chagas disease were treated immediately after being diagnosed. Thus, into the cohort of CCC I patients, 9 samples corresponded to individual samples (50%), 4 to a mixture of 2 patients (22.2%) and 5 to a mixture of 3 patients (27.8%). In the case of the cohort of IND patients, 15 samples corresponded to individual samples (38.5%), 16 to a mixture of 2 patients (41%) and 8 to a mixture of 3 patients (20.5%). For healthy donor’s cohort, 20 samples from subjects from non-endemic areas were assayed (all of them individual samples) together to 10 samples of donors from endemic areas, 6 of which corresponded to individual samples (60%) and 4 to a mixture of 2 subjects (40%).

### Isolation of peripheral blood mononuclear cells

Peripheral blood mononuclear cells (PBMCs) were isolated from twenty to thirty milliliter blood samples collected aseptically from each subject into EDTA-coated tubes. These cells were purified 16–18 hours after blood collection via a density gradient through centrifugation using Lymphoprep (Axis-Shield) following the previously described protocol [19]. The purified PBMCs were cryopreserved in liquid nitrogen until use, suspended in heat-inactivated fetal bovine serum (iFBS) with 10% dimethyl sulfoxide.

### Isolation of *T*. *cruzi* soluble antigens (*Tc*SA)

*T*. *cruzi* (SOL strain) soluble antigens (*Tc*SA), employed to perform *in vitro* stimulation of PBMCs from patients and healthy donors were extracted as previously described [15,20]. Briefly, mycoplasma-free LLC-MK2 line (CCL-7, Manassas, VA) were cultured at a concentration of 4x10^4^ cells/cm^2^ with RPMI-1640 medium (Gibco, Life Technologies) supplemented with 2 mM L-glutamine (Gibco), 10% iFBS and 50 μg/mL gentamicin (Thermo Fisher Scientific) at 37°C in a humidified atmosphere containing 5% CO_2_. Highly infective trypomastigote forms of the *T*. *cruzi* SOL strain (MHOM/ES/2008/SOL; DTU V) isolated from *T*. *cruzi* infected mice were employed to infect the semi-confluent monolayer of cells, at a parasite:cell ratio of 4:1, for 12 h. After 96–120 hours of infection, trypomastigote and amastigote forms present in the infected LLC-MK2 culture supernatants were collected, centrifuged at 1,258 rcf and washed twice with phosphate-buffered saline (PBS 1X). Subsequently, these parasites were resuspended, at a ratio of 1:1 (trypomastigote:amastigote) and a density of 1x10^6^ parasites/μL in lysis buffer (50 mM Tris-HCl at pH 7.4, 0.05% Nonidet P-40, 50 mM NaCl, 1 mM phenylmethylsulfonyl fluoride (PMSF), 1 μg/mL leupeptin), and sonicated three times with pulses of 50–62 kHz for 40 s with time intervals of 20 s. Finally, centrifugation at 6,700 rcf for 20 min at 4°C was applied to obtain total soluble protein extracts. The protein concentration of the extracts was determined using a micro bicinchoninic acid (BCA) protein assay kit (Thermo Fisher Scientific), and the protein profile was analyzed by SDS-PAGE after Coomassie blue staining. The antigenic and immunogenic capacities of *Tc*SA were tested by ELISA and lymphoproliferation assays using frozen splenocytes from *T*. *cruzi* chronically infected mice.

### Thawing and stimulation of peripheral blood mononuclear cells

Cryopreserved PBMCs were thawed and stimulated following the previously described laboratory protocol employed for detection of the production of cytokines, cytotoxic molecules, expression and coexpression of different inhibitory receptors in different subsets of cells [15,21,22]. Briefly, PBMCs were rapidly thawed in a 37°C water bath, transferred to a tube containing 10 mL of RPMI-1640 (supplemented with 2 mM L-glutamine, 10% iFBS and 50 μg/mL of gentamicin), centrifuged at 453 rcf for 10 min and suspended in 2 mL of supplemented RPMI-1640 medium. After that, the PBMCs (approximately 80% of viability) were plated at a concentration of 7.5–8.5x10^6^ cells/mL in 12-well plates and cultured for 4 h at 37°C in 5% CO_2_ to allow for the balance of basal gene expression under *in vitro* growth conditions. Finally, the PBMCs were stimulated with *Tc*SA (10 μg/mL) and cultured for 14–14.5 h at 37°C in 5% CO_2_.

### RNA isolation, quantification and quality analysis

Total RNA isolation from stimulated PBMCs was performed employing the RNeasy Plus Mini kit (Qiagen), eliminating the genomic DNA and obtaining mRNA enriched samples, according to the manufacturer’s recommendations. NanoDrop 1000 spectrophotometer (Thermo Fisher Scientific) and Qubit fluorometer (Invitrogen) were used to quantify and evaluate RNA purity. The quality of the extracted RNA was determined by analyzing its integrity by Bioanalyzer 2100 (Agilent Technologies) using RNA 6000 Nano kit (Agilent Technologies). All RNA samples included in this study had a high quality and integrity with an RNA integrity number (RIN) ranging from 7.4 to 10.

### Reverse transcription and high-throughput real-time quantitative PCR (RT-qPCR)

Reverse transcription was performed employing 2 μg of total RNA of each sample using High-Capacity cDNA Reverse Transcription kit (Applied Biosystems) following the manufacturer’s instructions. The resulting cDNA samples were stored at -20°C until use. Real time qPCR was performed using OpenArray plates (Thermo Fisher Scientific). PCR mixtures were prepared following the manufacturer’s instructions and were dispensed on OpenArray plates using the AccuFill system (Thermo Fisher Scientific). All reactions were performed in triplicate. The thermal cycle (95°C for 15 seconds, 60°C for 1 minute, for 40 cycles) and fluorescence detection were performed with the QuantStudio 12K Flex Real-Time PCR System (Thermo Fisher Scientific) according to the manufacturer’s protocol. Each custom plate contained FAM-MGB labeled Taqman probes specific to 106 immune response genes and 6 endogenous reference genes (indicated in italics in S1 Table) used as candidates for data normalization. All primers and probes were commercially available by Thermo Fisher Scientific. The complete list of the 112 genes and the corresponding Taqman assays are shown in S1 Table. Cq values produced by this platform are already corrected for the efficiency of the amplification [23].

### Data processing

The arithmetic average quantitative cycle (Cq) values for each qPCR run were exported from QuantStudio 12K Flex Real-Time PCR System, as Excel files, and were employed for data analysis. GenEx software (v.6, MultiD) was used for data preprocessing and normalization. Expression stability of all the 112 included genes was evaluated using GeNorm [24], NormFinder [25] and RefFinder [26] (heartcure.com.au), which integrates the four algorithms GeNorm [24], NormFinder [25], BestKeeper [27] and ΔCt method [28], in order to identify those showing highest stability across the whole dataset. After the stability score analyses, the 6 candidate reference genes included in the panel (*ACTB*, *B2M*, *GAPDH*, *HPRT1*, *PGK1*, *TBP*) showed low stability according to all the algorithms (indicated in gray bars in S1 Fig) and therefore were disregarded for normalization purposes. To improve normalization accuracy in this large dataset, three different reference genes were used for normalization [24,29]. The three most stably expressed genes, *STAT3*, *IL10RA* and *IFNAR* (indicated in bold in S1 Fig), all with geNorm M-values < 0.5 (which is the standard cutoff for reference genes selection) were selected as reference genes and used for normalization of the data set using GenEx software (v.6, MultiD) to obtain normalized relative quantity (NRQ) values.

### Enrichment analysis

Gene set enrichment analysis was performed on NRQ values of each group of patients as a whole using GSEA 4.1.0 computational method [30,31]. Canonical pathways gene sets derived from the BioCarta pathway database included in *C2*: *curated gene sets collection in Molecular Signatures Database (MSigDB)* [30,32] were selected for the enrichment analysis. The GSEA parameters were: permutations = 100,000, permutation type: phenotype (sample n > 7), enrichment statistic: weighted, metric for ranking genes: t-test, max size: 500, min size: 3.

### Statistical analyses

All statistical analyses were performed using IBM SPSS version 25 software (IBM Corporation, Armonk, NY, USA) and GraphPad Prism statistical package version 8 (GraphPad Software, San Diego, CA, USA). Kolmogorov-Smirnov and Shapiro-Wilk tests (α = 0.05) were used to check data normality and a two-tailed unpaired t-test or a two-tailed Mann-Whitney test, depending on data had or not a normal distribution, respectively, was applied to determine the statistical significance, considering *p* < 0.05 as statistically significant. Differentially-expressed genes between chronic cardiac Chagas disease patients (CCC I) and healthy donors (HD), as well as between CCC I patients and chronic Chagas disease patients with indeterminate form (IND) were identified using two parameters: the fold-change of gene expression (FC) and the statistical significance (*p*-value). FC was calculated as the ratio between the average gene expression level of the biological groups (CCC I/HD, CCC I/IND) as a way to indicate how many more (or less) times a particular gene is expressed in a biological group *versus* another. To be able to calculate the FC when the average gene expression level was 0 in one of the compared groups, number 0 was replaced by 0.000001 (this was applied for *CCR5* in the HD group and for *CLEC9A* in CCC I group). To display changes, volcano plots were made by plotting–log_10_ *p-*value (determined by a two-tailed unpaired t-test) on the y-axis, and log_2_ of FC on the x-axis. Genes passing both biological significance threshold (log_2_ of FC > 0.6 or < −0.6, corresponding to FC > 1.5 or < 0.66) and statistical significance threshold (–log_10_ *p* > 1.3, corresponding to *p* = 0.05), were marked in red and blue, attending to their upregulation and downregulation, respectively. Those genes were considered biologically relevant and used for further biological interpretation. In addition, in CCC I *versus* HD comparison, a stricter cutoff was also applied, log_2_FC > 1 or < -1, which corresponds to FC > 2 or < 0.5, respectively. Principal component analysis (PCA) was employed for multivariate analysis on NRQ values to determine the structure of the dataset. Differences in scores of plotted principal components between the groups were confirmed by an unpaired t-test or a Mann-Whitney test, as appropriate, using IBM SPSS 25 software.

## Results

### Identification of differentially expressed genes between cardiac Chagas disease patients and healthy subjects

In order to provide knowledge on the specific immune mechanisms triggered during chronic infection in Chagas disease patients with cardiac alterations, the expression pattern of different genes implicated in the immune response has been determined in these patients. In this way, the expression level of 106 immune system-related genes in response to *T*. *cruzi* soluble proteins has been determined in PBMCs from patients with chronic Chagas disease at the early stage of the cardiac phase. The resulting gene expression profile has been compared to that from healthy subjects from endemic and non-endemic regions of Chagas disease. Healthy subjects from endemic and non-endemic were considered as a single group regardless of their origin since previously reported principal component analysis did not show separation of samples into independent clusters as they had same behavior in terms of gene expression patterns [15].

Despite the comparative analyses performed among groups of patients were carried out as a whole and not as individual subject samples, the potential influence in the gene expression pattern of samples obtained from individual samples or from blended samples was also analysed. PCA analyses were performed considering samples from individual CCC I patients as a different group of the patients blended samples. The obtained results showed that all CCC I samples conserved the same distribution independently whether they came from individual patients (50% of the samples) or they had been blended (S2 Fig). The same level of gene expression in the CCC I samples was supported by two-tailed unpaired t-test, which showed that there were no statistically significant differences between the scores obtained in the two groups for each principal component (S2A Fig). By contrast, as expected, statistically significant differences were observed when data from both single and blended samples from CCC I patients were compared to the gene expression level of HD (S2B Fig) (*p* < 0.0001 in both).

**Fig 1:**
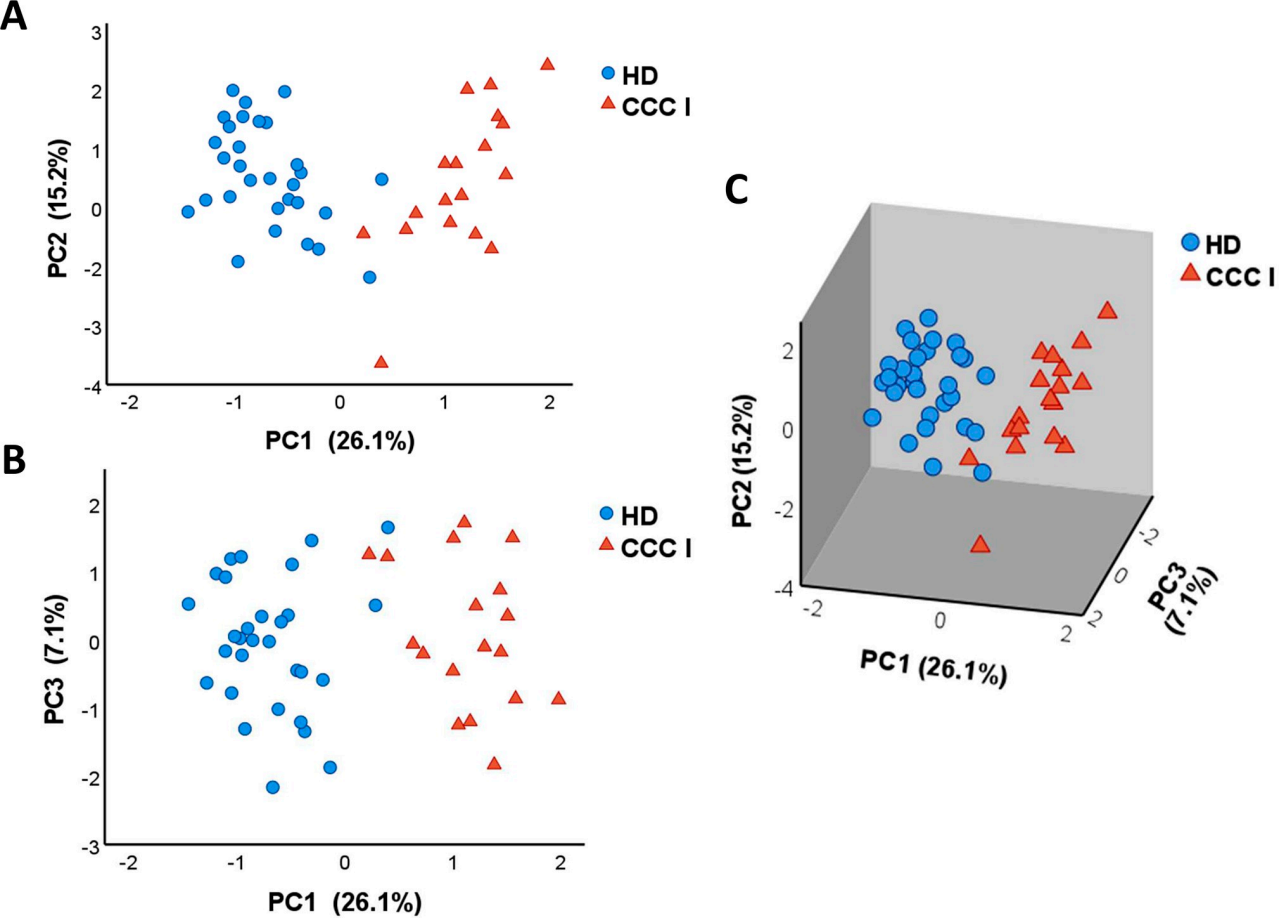
Principal component analysis (PCA) of gene expression profiles in cardiac Chagas disease and healthy subjects. PCA was applied on NRQ values of expression level of 106 analyzed genes from cardiac Chagas disease patients (CCC I, orange triangles) and healthy donors (HD, blue circles). (A) PCA score plot of Principal Components 1 (PC1) and 2 (PC2) on the x and y-axis, respectively. (B) PCA score plot of Principal Components 1 (PC1) and 3 (PC3) on the x and y-axis, respectively. (C) 3D graphic representing the three Principal Components 1, 2 and 3 (PC1, PC2 and PC3). The proportion of total variance related to each principal component is given as a percentage and indicated on the axis next to the corresponding principal component.

With the aim of determining gene expression profiles in all samples of cardiac Chagas disease patients (Kuschnir I) (CCC I; n = 18) and healthy donor subjects (HD; n = 30), principal component analysis (PCA) was performed employing normalized relative quantities (NRQ). Thus, four components were extracted and the scores obtained for each of them were plotted on graphs (Fig 1). Principal components 1 (PC1) and 2 (PC2), which explain 26.1% and 15.2% of the variance, respectively, are shown in Fig 1A, and principal components 1 and 3 (PC3), responsible for 7.1% of the variance, are shown in Fig 1B. The three principal components were also represented in the same graph, using a 3D scatter plot (Fig 1C). Based on PC1 of the PCA analyses, CCC I patients and healthy donors formed two independent subsets (Fig 1). The differences in the scores were confirmed by a two-tailed unpaired t-test which highlighted the existence of a statistically significant different expression profile in CCC I patients compared to healthy donors (*p* < 0.0001), focused on the genes correlated with PC1 (S2 Table). PC1 depends on the expression of 36 genes, which correlate with a high factor loading. 26 out of the referred 36 genes (*FAS*, *BTLA*, *CSF2*, *CASP3*, *CD274*, *IL12A*, *IL12RB1*, *IL7*, *CD83*, *IL12RB2*, *CD40*, *IL6*, *IL2RA*, *TBX21*, *IL23A*, *STAT1*, *CSF1*, *IFNG*, *BCL2*, *CD40LG*, *CD69*, *TNF*, *IL5RA*, *CD80*, *IL2RG* and *CD2*) are positively correlated showing a factor loading greater than 0.6. By contrast, 10 genes (*TGFBR1*, *CCR1*, *ITGB2*, *IL18*, *IFNGR2*, *IFNGR1*, *CD86*, *ITGAX*, *HAVCR2* and *IL17RA*) negatively correlate with PC1 (factor loading lower than -0.6) (S2 Table). The same statistical analysis was also applied to the scores obtained in CCC I and HD for the rest of the extracted principal components, although data indicated that these components did not contribute with statistical significance to the differences observed between both groups of subjects (PC2 *p* = 0.720; PC3 *p* = 0.629; PC4 *p* = 0.073).

**Fig 2:**
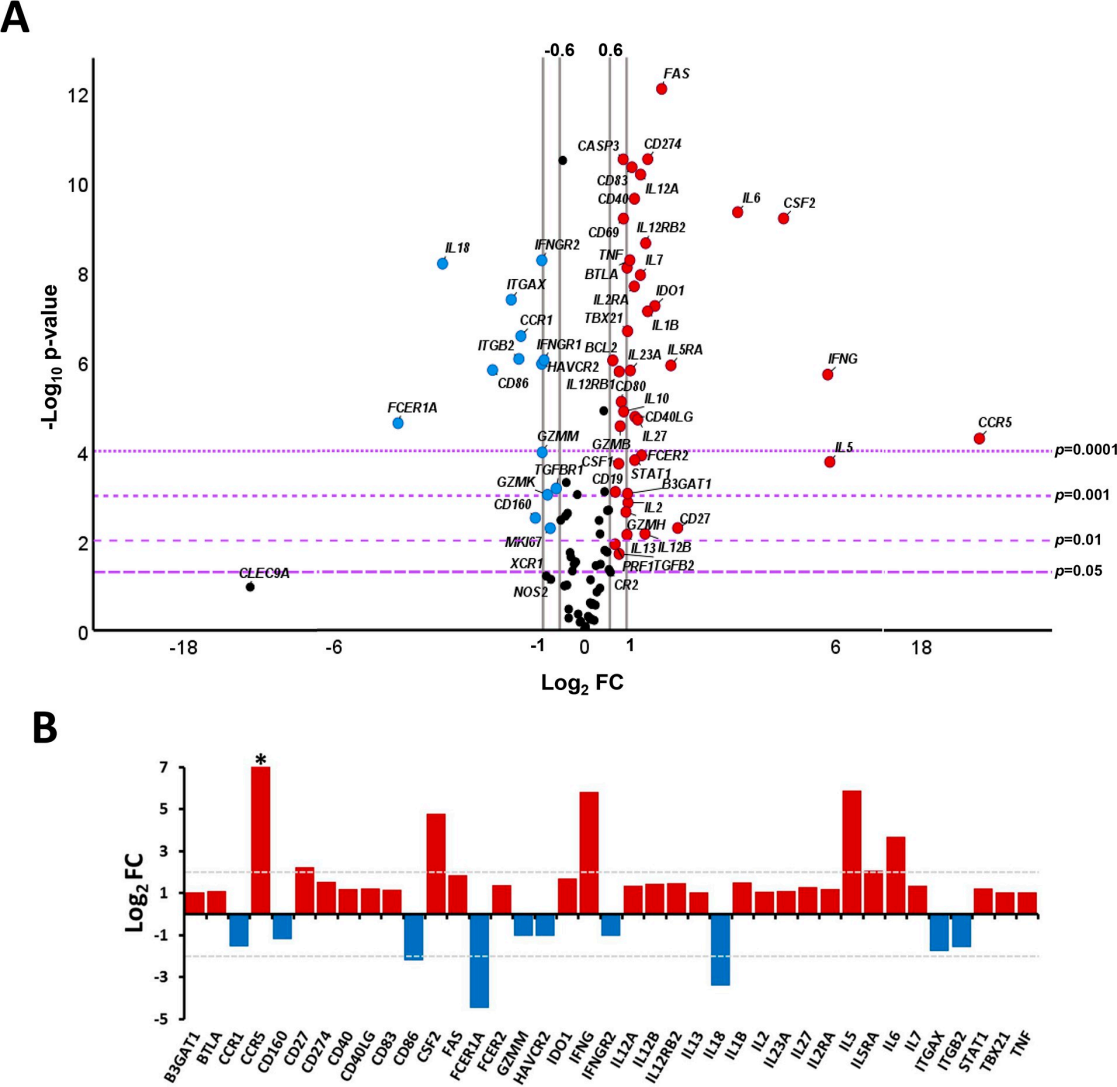
Differential gene expression level in cardiac Chagas disease patients *versus* healthy donors. (A) Volcano plot showing the differential expression level of the 106 analyzed genes in chronic Chagas disease patients with cardiac alterations (CCC I) (n = 18) and healthy donors (HD) (n = 30). The x-axis represents log_2_ of expression fold-change between CCC I and HD (Log_2_FC), where FC is calculated as the ratio between the average gene expression in the two groups (CCC I/HD). The y-axis corresponds to the statistical significance, expressed as the negative logarithm of the *p*-value (-Log_10_ *p*-value). The purple horizontal lines indicate the cut-off for the statistical significance (corresponding to *p* = 0.05, *p* = 0.01, *p* = 0.001 and *p* = 0.0001). The grey vertical lines represent the log_2_FC of -0.6 and 0.6 (corresponding to FC of 0.66 and 1.5, respectively) and log_2_FC of −1 and 1 (corresponding to FC of 0.5 and 2, respectively) used as biological thresholds to identify differentially expressed genes. Negative values correspond to downregulated genes (blue dots) and positive values to upregulated genes (red dots) in CCC I compared to HD subjects. Black dots represent non-differentially expressed genes between CCC I and HD subjects. *Since the *CCR5* gene did not show any detectable expression in the HD group, to apply the formulas, the value 0 was replaced by 10^−6^, resulting in a Log_2_FC value of 19.35. (B) Expression level of 39 genes differentially expressed in cardiac Chagas disease patients *versus* healthy donors. Each plot bar corresponds to the gene referred at the bottom of the graph (x-axis). The y-axis represents log_2_ of expression fold-change (Log_2_FC) for each gene. Positive values (red bars) indicate upregulated genes and negative values (blue bars) indicate downregulated genes in CCC I compared to HD subjects. The grey horizontal lines highlight the genes with the largest differences in expression of at least more than four or less than a quarter-fold (Log_2_FC > 2; Log_2_FC < -2). *Since the CCR5 gene did not show any detectable expression in the HD group, *to apply the formulas*, *the value 0 was replaced by 10*^*−6*^, *resulting in a Log*_*2*_*FC value of 19*.*35*.

To quantify the differential expression level of the genes under study in CCC I patients and healthy donors, the fold-change was calculated as the ratio of expression between CCC I and HD (Fig 2). The obtained results indicated that 55 out of the 106 analyzed genes showed a statistically significant differential gene expression level being 41 of them upregulated and 14 downregulated in CCC I *versus* HD (-log_10_ *p*-value > 1.3, equivalent to a *p*-value < 0.05 and log_2_FC > 0.6 or < -0.6). When a stricter threshold was set (Log_2_FC > 1 or < -1), 39 genes showed to be differentially expressed being 29 of them upregulated with statistically significance in CCC I (*B3GAT1*, *BTLA*, *CCR5*, *CD27*, *CD274*, *CD40*, *CD40LG*, *CD83*, *CSF2*, *FAS*, *FCER2*, *IDO1*, *IFNG*, *IL12A*, *IL12B*, *IL12RB2*, *IL13*, *IL1B*, *IL2*, *IL23A*, *IL27*, *IL2RA*, *IL5*, *IL5RA*, *IL6*, *IL7*, *STAT1*, *TBX21* and *TNF*) showing at least twice the expression level detected in healthy donors (Log_2_FC > 1, Fig 2A red dots). The expression level of 10 genes (*CCR1*, *CD160*, *CD86*, *FCER1A*, *GZMM*, *HAVCR2*, *IFNGR2*, *IL18*, *ITGAX* and *ITGB2*) showed to be downregulated in CCC I subjects with at least less than a half the expression level detected in healthy subjects (Log_2_FC < -1, Fig 2A blue dots). All the differences observed in the referred 39 genes were statistically significant (Fig 2A), identifying 29 out of 39 with *p*-values lower than 0.0001, 5 with *p*-values lower than 0.001 and 5 genes with *p*-values lower than 0.01.

By analysing the magnitude of the changes in the gene expression of the 39 differentially expressed genes between CCC I and HD subjects, 10 genes showed strong differences (at least twice the established cut-off of log_2_FC > 1 and < −1, Fig 2A as observed in the data bar plot representation (Fig 2B). Thus, 7 of them particularly upregulated (*CCR5*, *CD27*, *CSF2*, *IFNG*, *IL5*, *IL5RA* and *IL6*) and the other 3 downregulated (*CD86*, *FCER1A* and *IL18*) in CCC I patients *versus* HD. Thus, *CD27* and *IL5RA* genes showed to be at least 4 times higher expressed in CCC I than in HD (Log_2_FC > 2) and *IL6* and *CSF2* genes greater than 12 and 27 times, respectively (Log_2_FC of 3.7 and 4.8). The largest differences were detected for *IFNG* and *IL5* genes (log_2_FC = 5.8), which showed approximately 56 times higher expression level in CCC I patients than in healthy subjects. No expression of *CCR5* gene was detected in any healthy donor although it showed to be highly expressed in the CCC I patients. By contrast, the expression level of *CD86* and *IL18* genes in CCC I patients (Log_2_FC lower than -2 and -3, respectively) was a quarter and an eighth of the expression level observed in HD (FC < 0.25 and 0.125, respectively). The greatest downregulated gene was *FCER1A* (Log_2_FC = -4.5), which showed an expression level up to 20 times lower in CCC I patients than in healthy subjects (FC = 0.05).

### Differential expression of immune response genes in chronic Chagas disease patients with cardiac compromise *versus* asymptomatic

In order to evaluate how the antigen specific immune response generated against *T*. *cruzi* in patients with chronic Chagas disease is involved in disease progression, we compared the expression pattern of the 106 selected genes involved in the immune response in patients with Chagas disease in the early cardiac phase (CCC I, n = 18) with that from patients in the indeterminate phase (IND, n = 39). Thus, a principal component analysis was applied to the set of NRQ values of the 106 genes under study from both patient groups to determine their structure and expression similarities between IND and CCC I subjects (S3 Fig). Principal component 1 (PC1) and principal component 2 (PC2) did not show striking differences in gene expression between the two groups of patients (two-tailed unpaired t-test PC1 *p* = 0.263 and PC2 *p* = 0.387), (S3A Fig). Representation of principal component 4 (PC4) showed a trend towards positive scores for PC4 (represented on the y-axis) in CCC I patients (S3B Fig). These differences in scores for PC4 between both groups of individuals was confirmed by a two-tailed Mann–Whitney test that revealed the existence of a statistically significant differential expression profile in CCC I *versus* IND patients (*p* = 0.008). Thus, 3 genes were correlated to PC4 with high factor loading, two of them were positively correlated to this component (*IL1B* and *CD58*) and one (*SELL*) negatively correlated. A heatmap determined by GSEA considering the 100 genes which exhibit the greatest differences in the expression level between the samples from CCC I and IND patients (S4 Fig) revealed a tendency in CCC I towards lower expression levels in approximately half of the genes represented. Optically, in contrast, approximately 10% of the genes showed overall higher level of expression in CCC I patients compared to IND patients (S4 Fig).

**Fig 3:**
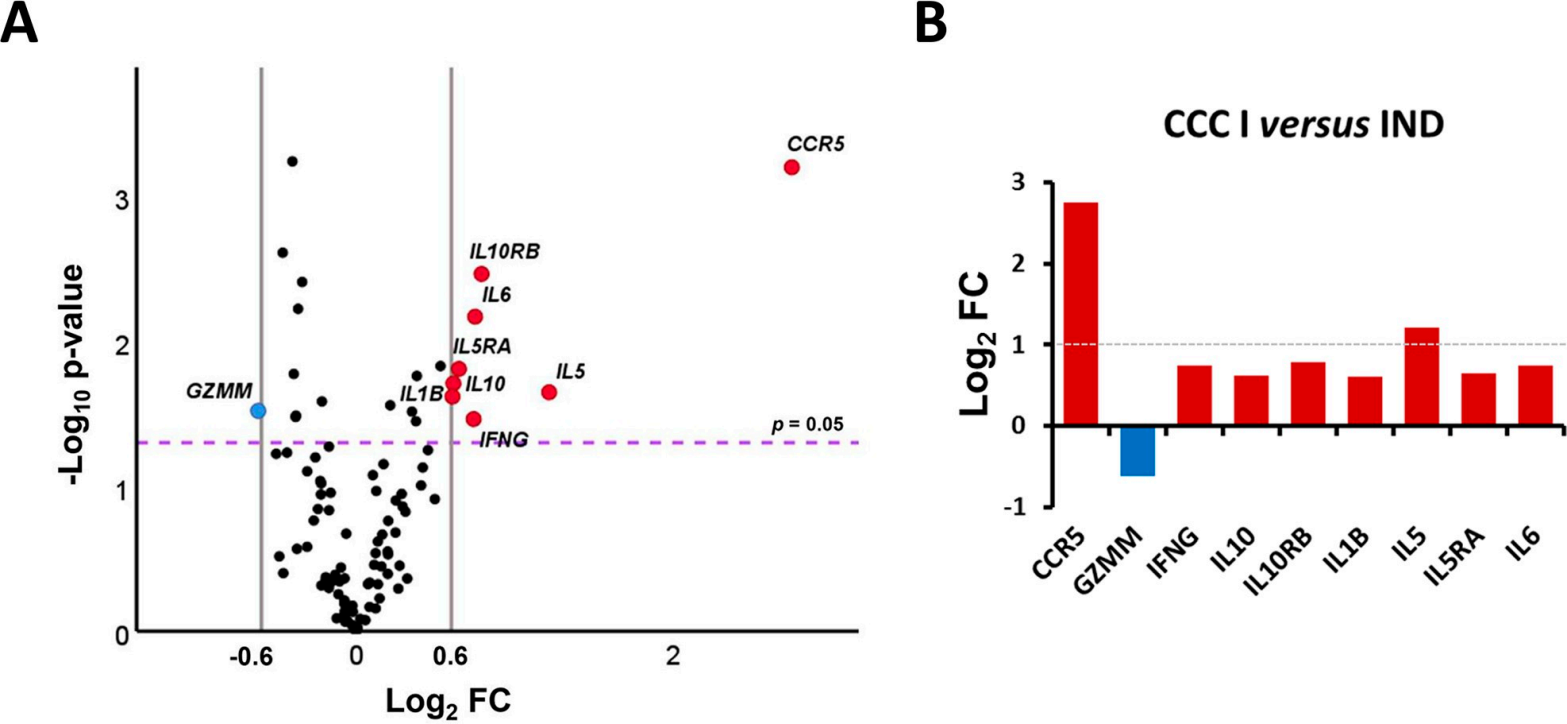
Differential gene expression level in cardiac Chagas disease patients *versus* healthy donors. (A) Volcano plot representing the differential gene expression level of the 106 analyzed genes in chronic Chagas disease patients with indeterminate form (IND) (n = 39) and patients with cardiac alterations (CCC I) (n = 18). The x-axis represents log_2_ of expression fold-change between CCC I and IND (Log_2_FC), where FC is calculated as the ratio between the average gene expression in two groups (CCC I/IND). The y-axis corresponds to the statistical significance, expressed as the negative logarithm of the *p*-value (-Log_10_ *p*-value). The purple horizontal line indicates the cut-off for the statistical significance (corresponding to *p* = 0.05). The grey vertical lines represent the log_2_FC of −0.6 and 0.6 (corresponding to FC of 0.66 and 1.5, respectively) used as biological thresholds to identify differentially expressed genes. Negative values correspond to downregulated genes (blue dots) and positive values to upregulated genes (red dots) in CCC I compared to IND patients. Black dots represent non-differentially expressed genes between CCC I and IND patients. (B) Relative expression level of 9 genes differentially expressed in cardiac *versus* indeterminate Chagas disease patients. Each plot bar corresponds to the gene referred at the bottom of the graph (x-axis). The y-axis represents the log_2_ of expression fold-change (Log_2_FC) for each gene. Positive values (red bars) indicate upregulated genes and negative values (blue bars) indicate downregulated genes in CCC I compared to IND patients. The grey horizontal line indicates the cut-off to highlight the genes with the greatest differences in expression (Log_2_FC > 1).

To identify and analyse in detail the genes that exhibited a differential expression level in CCC I *versus* IND and could be involved in disease progression, a comparative analysis was performed based on the fold-change of gene expression (FC) and statistical significance (Fig 3). The results showed that 9 out of the 106 genes studied were differentially expressed between CCC I and IND patients with statistical significance. Eight of these genes (*CCR5*, *IFNG*, *IL10*, *IL10RB*, *IL1B*, *IL5*, *IL5RA* and *IL6*) were upregulated at least more than one and a half times in CCC I *versus* IND patients with Log_2_FC > 0.6 (corresponding to FC > 1.5, Fig 3A red dots), whereas only *GZMM* was found to be downregulated in CCC I patients when compared to IND patients with Log_2_FC < -0.6, corresponding to at least less than one third times (FC < 0.66, Fig 3A blue dot). As observed in Fig 3A, the observed differences in the expression level were statistically significant for all 9 genes with a -log10 *p*-value higher than 1.3 (*p*-value < 0.05). The magnitude of the changes between CCC I and IND subjects are shown in Fig 3B, revealing that 2 out of the 9 differentially expressed genes had a particular upregulation in CCC I patients with respect to IND. Thus, *IL5* and *CCR5* exhibited the highest gene expression differences (log_2_FC = 1.2 and 12.75, respectively) which means an upregulation of these genes more than double and more than six times, respectively, in CCC I patients than in IND.

**Fig 4:**
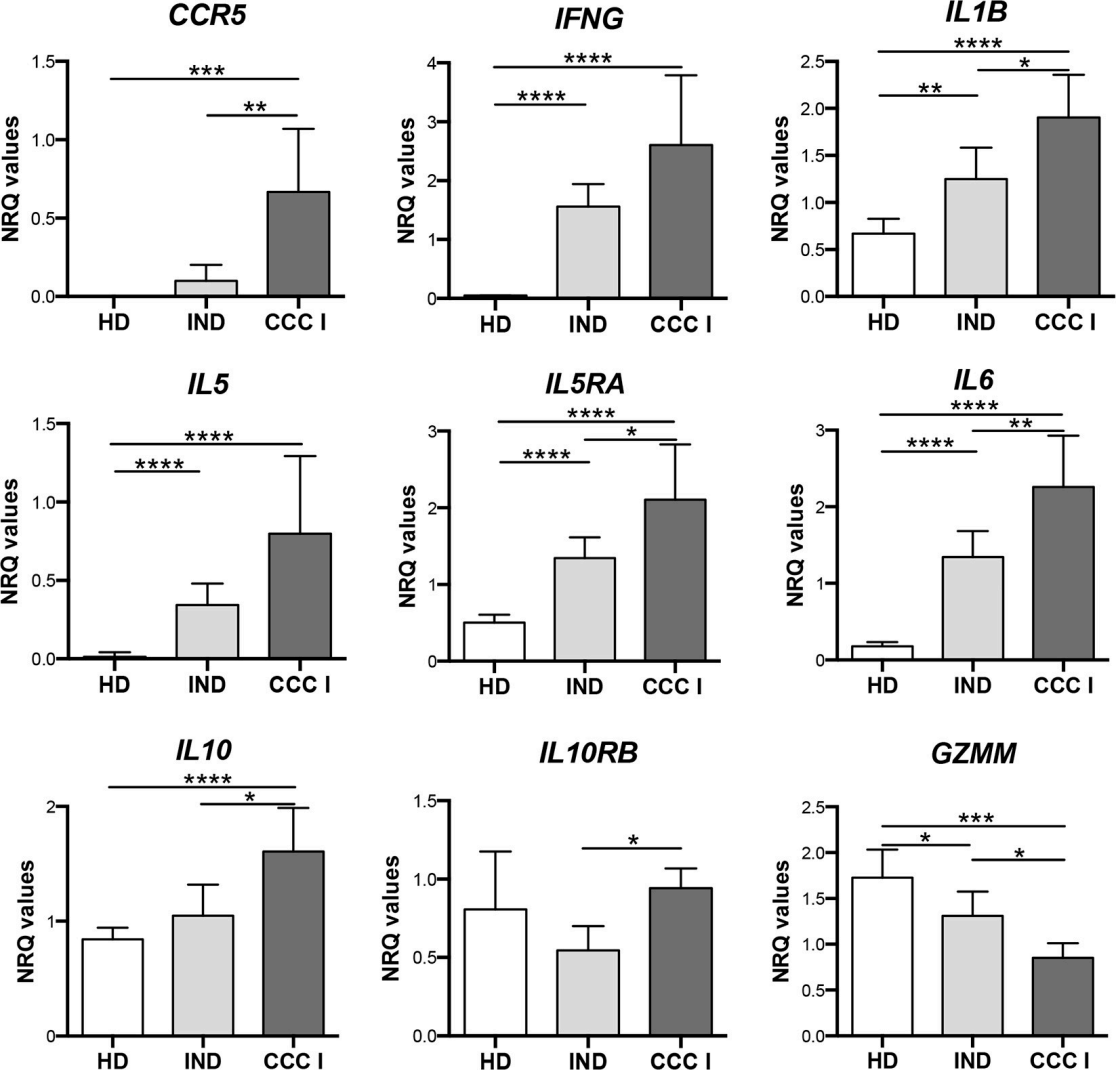
Comparison of gene expression level in Chagas disease patients (cardiac and indeterminate) and healthy donors. Expression level of *CCR5*, *IFNG*, *IL1B*, *IL5*, *IL5RA*, *IL6*, *IL10*, *IL10RB* and *GZMM* genes measured as mean Normalized Relative Quantities (NRQ) in patients with chronic Chagas disease with indeterminate form (IND), patients with cardiac alterations (CCC I) and healthy donors (HD). Statistically significant differences determined by two-tail Mann-Whitney test or two-tailed unpaired t-test, as appropriate, are indicated (* *p* < 0.05, ** *p* < 0.01, *** *p* < 0.001 and **** *p* < 0.0001).

To analyze the behavior that the 9 differentially expressed genes between CCC I and IND had through the course of the infection, it was also taken into consideration their expression level in healthy donors. As observed in the bar plots shown in Fig 4, the results revealed that expression of *CCR5*, *INFG*, *IL1B*, *IL5*, *IL5RA*, *IL6*, *IL10* and *IL10RB* genes increased and of *GZMM* decreased with statistically significance as the disease progressed. Thus, the expression of 3 genes (*IL1B*, *IL5RA*, *IL6*) increased and 1 gene (*GZMM*) decreased with statistically significant as the disease progressed since there were differences between CCC I and IND and also between IND and HD. Differences in the expression level of these 4 genes were also observed between CCC I and HD. Statistically significant differences in the expression level of *INFG* and *IL5* were observed between CCC I and HD and also between IND and HD although they were not observed between CCC I and IND. Differences in the expression level between CCC I and IND were detected for *CCR5*, *IL10* and *IL10RB*, which were also important between CCC I and HD for *CCR5* and *IL10*.

A summary of the differentially expressed genes in CCC I *versus* HD as well as IND *versus* HD and CCC I *versus* IND that exhibited a log_2_FC > 0.6 or < -0.6 and log_2_FC > 1 or log_2_FC < -1 were compiled in Table 2. The comparative analyses revealed that 47 genes were differentially expressed (35 genes upregulated and 12 downregulated) in both CCC I and IND patients *versus* HD. Among these 47 differentially expressed genes, 21 were particularly upregulated (log_2_FC > 1) and 8 downregulated (log_2_FC < -1) in both CCC I and IND patients *versus* HD (Table 2, genes in bold face). Furthermore, 6 genes were upregulated (*CCR5*, *CD19*, *IL10*, *IL13*, *PRF1* and *TGFB2*) and 2 downregulated (*GZMK* and *GZMM*) solely in CCC I *versus* HD and 8 genes showed to be upregulated (*CD28*, *CD3E*, *CR2*, *GATA3*, *ICOS*, *IL2RG*, *LAG3* and *TNFSF10*) and 4 downregulated (*CLEC9A*, *GZMA*, *ICAM1* and *XCR1*) solely in IND patients *versus* HD (Table 2).

**Table 2:** Differentially expressed genes in chronic Chagas disease patients (Log_2_FC >0.6 or <-0.6 and *p* <0.05).

Upregulated			Downregulated		
**IND *versus* HD**	**CCC I *versus* HD**	**CCC I *versus* IND**	**IND *versus* HD**	**CCC I *versus* HD**	**CCC I *versus* IND**
*B3GAT1*	***B3GAT1***		***CCR1***	***CCR1***	
***BCL2***	*BCL2*		*CD160*	***CD160***	
***BTLA***	***BTLA***		***CD86***	***CD86***	
*CASP3*	*CASP3*		***CLEC9A***		
	***CCR5***	***CCR5***	***FCER1A***	***FCER1A***	
	*CD19*		*GZMA*		
***CD27***	***CD27***			*GZMK*	
***CD274***	***CD274***			***GZMM***	*GZMM*
*CD28*			*ICAM1*		
*CD3E*			***IFNGR1***	*IFNGR1*	
***CD40***	***CD40***		***IFNGR2***	***IFNGR2***	
***CD40LG***	***CD40LG***		***IL18***	***IL18***	
*CD69*	*CD69*		***ITGAX***	***ITGAX***	
*CD80*	*CD80*		***ITGB2***	***ITGB2***	
*CD83*	***CD83***		*MKI67*	*MKI67*	
*CR2*			*TGFBR1*	*TGFBR1*	
***CSF1***	*CSF1*		***HAVCR2***	***HAVCR2***	
***CSF2***	***CSF2***		***XCR1***		
***FAS***	***FAS***				
*FCER2*	***FCER2***				
*GATA3*					
*GZMB*	*GZMB*				
*GZMH*	*GZMH*				
*ICOS*					
***IDO1***	***IDO1***				
***IFNG***	***IFNG***	*IFNG*			
	*IL10*	*IL10*			
		*IL10RB*			
***IL12A***	***IL12A***				
***IL12B***	***IL12B***				
*IL12RB1*	*IL12RB1*				
***IL12RB2***	***IL12RB2***				
	***IL13***				
*IL1B*	***IL1B***	*IL1B*			
***IL2***	***IL2***				
*IL23A*	***IL23A***				
***IL27***	***IL27***				
*IL2RA*	***IL2RA***				
*IL2RG*					
***IL5***	***IL5***	***IL5***			
***IL5RA***	***IL5RA***	*IL5RA*			
***IL6***	***IL6***	*IL6*			
***IL7***	***IL7***				
*LAG3*					
	*PRF1*				
***STAT1***	***STAT1***				
***TBX21***	***TBX21***				
	*TGFB2*				
***TNF***	***TNF***				
*TNFSF10*					

IND: patients with indeterminate form; CCC I: patients with cardiac alterations; HD: healthy donors. Bold letters indicate those genes with Log_2_FC > 1 or < -1.

### Immune pathways enriched in chronic Chagas disease patients

**Fig 5:**
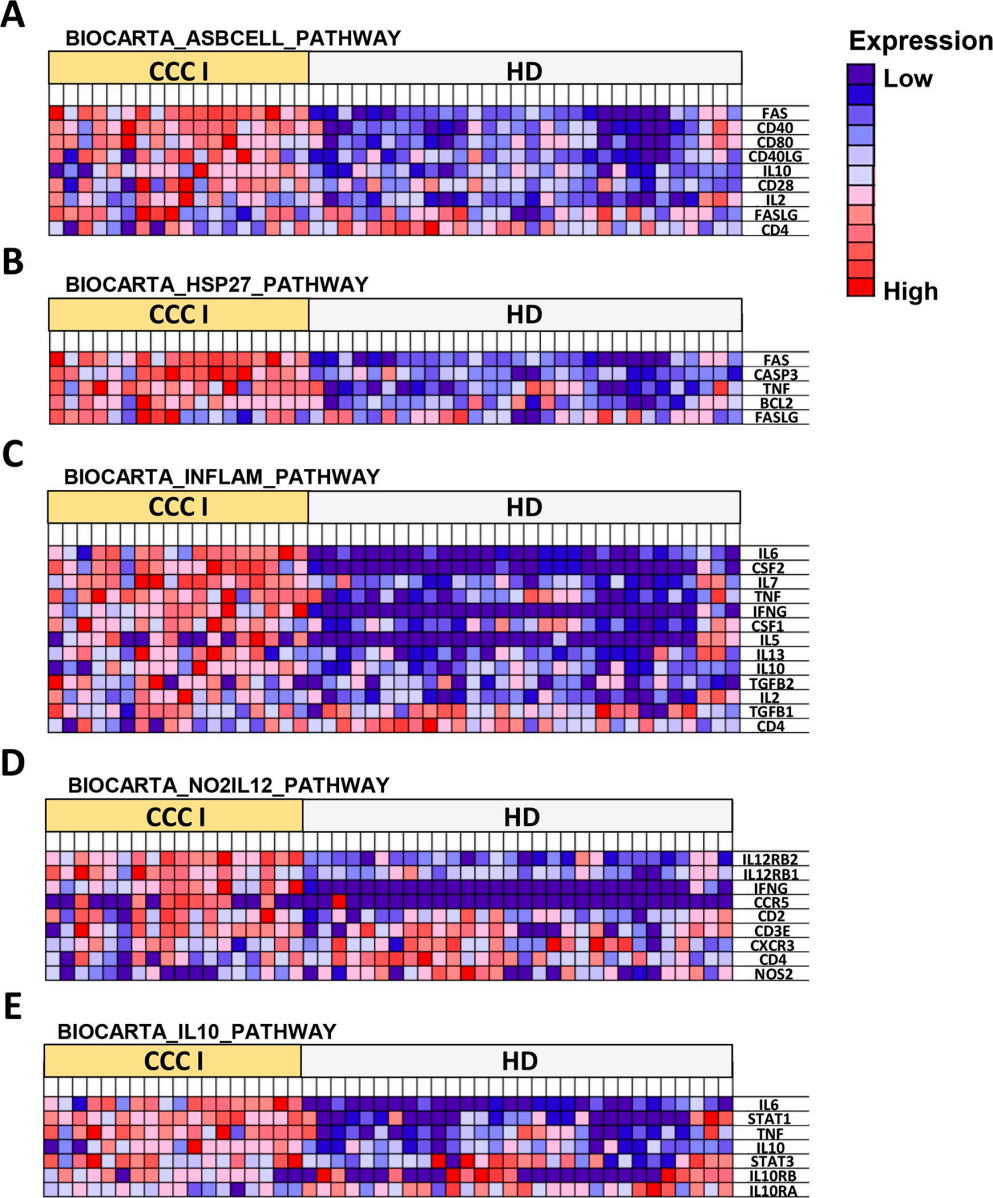
Gene set enrichment analysis (GSEA) in cardiac Chagas disease patients *versus* healthy donors. GSEA heatmaps of representative gene sets from (A) Antigen dependent B cell activation pathway (BIOCARTA_ASBCELL_PATHWAY), (B) Stress induction of HSP regulation (BIOCARTA_HSP27_PATHWAY), (C) Cytokines and inflammatory response (BIOCARTA_INFLAM_PATHWAY), (D) NO2-dependent IL12 pathway in NK cells (BIOCARTA_NO2IL12_PATHWAY), and (E) IL-10 anti-inflammatory signaling pathway (BIOCARTA_IL10_PATHWAY) enriched in CCC I patients *versus* HD subjects. The values of the expression level of each gene are represented as colors, ranging from dark red to dark blue, based on the highest and lowest normalized relative quantity (NRQ) value of each gene, respectively. Parameters set for GSEA were: *Molecular Signatures Database* (MSigDB) BioCarta gene set collection, permutations = 100,000, permutation type: phenotype, enrichment statistic: weighted, metric for ranking genes: t-test, max size: 500, min size: 3.

To determine the immunological pathways involved in the observed gene-expression differences between CCC I and healthy donors, gene set enrichment analysis (GSEA) was performed using NRQ values of genes from these subjects. For this purpose, the *Molecular Signatures Database* (MSigDB) BioCarta gene set collection [30,32] was employed. As observed in the heatmaps shown in Fig 5, GSEA revealed a consistent upregulation of genes that participate in several relevant immunological pathways in CCC I patients compared to HD. The gene sets enriched in these patients were shown to be involved in the antigen-dependent B cell activation (BIOCARTA_ASBCELL_PATHWAY, Fig 5A), stress induction of HSP regulation (BIOCARTA_HSP27_PATHWAY, Fig 5B), cytokine and inflammatory responses (BIOCARTA_INFLAM_PATHWAY, Fig 5C), NO2-dependent IL12 pathway in NK cells (BIOCARTA_NO2IL12_PATHWAY, Fig 5D) and IL-10 anti-inflammatory signaling pathway (BIOCARTA_IL10_PATHWAY, Fig 5E), with a normalized enrichment score (NES) of 1.16, 1.37, 1.4, 0.86, and 1.24, respectively.

The immune pathways enriched in CCC I and in asymptomatic Chagas disease patients *versus* healthy donors, respectively, were subsequently compared according to GSEA and taking into consideration the upregulated genes (Table 3). Thus, to complete the analysis, the upregulated genes with log_2_FC higher than 0.6 and those correlated with principal component 1 (factor loading > 0.6) were considered. Particularly, the enrichment of the "Antigen dependent B cell activation" BioCarta pathway (indicated in green in Table 3) in IND and CCC I patients was driven by the upregulation of *CD28* (DEG only in IND), *CD40*, *CD40LG*, *FAS*, *IL2* and *IL10* (DEG only in CCC I) genes. 4 genes involved in the "Stress induction of HSP regulation" pathway (*FAS*, *CASP3*, *BCL2* and *TNF*) were upregulated in IND and CCC I *versus* HD. The enrichment of the "Cytokines and inflammatory response" pathway (Table 3, blue) was a consequence of the upregulation of 8 genes (*IL2*, *TNF*, *CSF1*, *CSF2*, *IFNG*, *IL5*, *IL6* and *IL7*) in IND and the upregulation of the same 8 genes, plus *IL10*, *IL13* and *TGFB2* in CCC I patients. In addition, 4 genes of the "NO2-dependent IL-12 pathway in NK cells" (Table 3, yellow) were found to be upregulated in IND and CCC I patients *versus* HD subjects (*IFNG*, *CD2*, *IL12RB1*, *IL12RB2*) and *CCR5* in CCC I *versus* HD. Similarly, GSEA identified “IL-10 anti-inflammatory signaling” (Table 3, purple) as an enriched pathway in these patients, a consequence of upregulation of *TNF*, *IL6*, *IL10* (DEG only in CCC I) and *STAT1* when compared to the gene expression level detected in healthy subjects.

**Table 3:** Genes upregulated in Chagas disease patients *versus* healthy donors that were involved in BioCarta pathways.

Gene	BioCarta pathways					Log_2_FC >0.6; *p* < 0.05		Factor Loading for PC1 > 0.6	
						IND vs HD	CCC I vs HD	IND vs HD	CCC I vs HD
***CD28***						**✓**		**✓**	
***CD40***						**✓**	**✓**	**✓**	**✓**
***CD40LG***						**✓**	**✓**	**✓**	**✓**
***CD80***						**✓**	**✓**		**✓**
***FAS***						**✓**	**✓**	**✓**	**✓**
***IL2***						**✓**	**✓**		
***CASP3***						**✓**	**✓**	**✓**	**✓**
***BCL2***						**✓**	**✓**	**✓**	**✓**
***TNF***						**✓**	**✓**	**✓**	**✓**
***CSF1***						**✓**	**✓**	**✓**	**✓**
***CSF2***						**✓**	**✓**		**✓**
***IFNG***						**✓**	**✓**	**✓**	**✓**
***IL5***						**✓**	**✓**		
***IL6***						**✓**	**✓**		**✓**
***IL7***						**✓**	**✓**	**✓**	**✓**
***IL10***							**✓**		
***IL13***							**✓**		
***TGFB2***							**✓**		
***CCR5***							**✓**		
***CD2***								**✓**	**✓**
***IL12RB1***						**✓**	**✓**	**✓**	**✓**
***IL12RB2***						**✓**	**✓**	**✓**	**✓**
***STAT1***						**✓**	**✓**	**✓**	**✓**

BioCarta Antigen Dependent B Cell Activation (BIOCARTA_ASBCELL_PATHWAY; green), Stress Induction of HSP Regulation (BIOCARTA_HSP27_PATHWAY; orange), Cytokines and Inflammatory Response (BIOCARTA_INFLAM_PATHWAY; blue), NO2-dependent IL 12 Pathway in NK cells (BIOCARTA_NO2IL12_PATHWAY; yellow) and IL-10 Anti-inflammatory Signaling Pathway (BIOCARTA_IL10_PATHWAY; purple) were enriched in IND and/or CCC I *versus* HD according to GSEA analysis. FC: fold-change, FL: factor loading, PC1: Principal component 1. The ticks indicate the genes that meet the criteria established in the header of each column.

## Discussion

As a consequence of Chagas disease, more than two million people are going to develop cardiac involvement which may compromise their life requiring in many cases pacemaker implant. Most commonly, chronic cardiac Chagas disease (CCC) develops after decades of the indeterminate (IND) form of the disease, reflecting the establishment of a new state of imbalance between the parasite and the host immune response. The factors that drive progression of the disease are not fully understood, although longitudinal studies have highlighted several possible risk factors such as parasite strain, exposure to reinfection, age, genetic background, the severity of acute infection, nutritional status, and other concomitant diseases of the patients [33]. Regarding host genetics, susceptibility to CCC may be the result of genetic polymorphisms of chemokines, cytokines and genes involved in the immune response [12,34] which result in a different level of gene expression.

Given the existence of differential susceptibility to Chagas disease and in the development of its chronic form, together with the lack of biomarkers of disease progression and effective treatment in the chronic phase, it is essential to characterize the biological processes and immunological pathways involved in the establishment of chronic cardiac Chagas disease. Most research carried out in the field has focused on elucidating the role and importance of individual cell subsets or activation profiles to control *T*. *cruzi* infection, and their association to the chronic pathology of Chagas disease [35]. However, these approaches do not enable the integration of the processes involved anti-*T*. *cruzi* immunity, taking into consideration the complexity of the interconnected and interdependent pathways of the immune system [35]. To this end, in this study, a high-throughput real-time qPCR analysis has been applied to determine simultaneously global changes in antigen-specific gene expression profiles of 106 immune system-related genes in CCC I patients.

Cytokines play an essential role in immune response regulation. In particular, chemokines have a crucial function in the control of leukocyte migration during the host response to infectious processes [36,37]. In addition, inflammatory cytokines and chemokines have been implicated in the generation of inflammatory infiltrates and tissue damage [34]. The results obtained in the present paper shown the relevance of *IFNG* and *IL5* in the *T*. *cruzi*-specific immune response of CCC I patients. These two chemokines were strongly upregulated, with up to 56-fold and 58-fold higher expression, respectively, in cardiac I patients compared to healthy subjects. *IFNG* plays a key role in the control of *T*. *cruzi* infection by participating in the differentiation of CD4^+^ Th1 and CD8^+^ T cells and leading to intracellular elimination of parasites [38,39]. In contrast, *IFNG* also has a central pathogenic role in Chagas disease by inducing heart damage through a variety of mechanisms [40]. It was also the most upregulated cytokine when we performed this study in IND patients *versus* healthy subjects [15]. Thus, while the indeterminate form is associated with a balanced immune response with a robust cellular response and high expression of *IFNG* [41], there is controversy in the cardiac form. Some authors associate its increased expression in the cardiac form with the establishment of an unbalanced inflammatory response [41]. On the contrary, other authors associate its increased expression with poor responses characterized by monofunctional *T*. *cruzi*-specific T cells that produce IFN-γ [42,43] and also due to the death of effector cells [44]. The high levels of *IFNG* detected in this study in CCC I patients could indicate that, in this initial cardiac phase, effector cells still maintain their functional capacity. Regarding *IL5*, the upregulation detected in these CCC I patients *versus* healthy donors suggests that in this cardiac phase there is also an anti-inflammatory Th2 response, as it was observed in IND patients who showed high expression levels of *IL5* gene [15]. In addition, the expression level of other genes such as *CD27*, *CSF2*, *IL5RA* and *IL6* were also elevated in CCC I patients. Similarly, genes coding for CD27, CSF2 and IL6 were also markedly upregulated in patients with indeterminate form compared to healthy subjects [15]. An elevated level of IL6 produced by CD8^+^ T cells in response to stimulation with immunogenic peptides contained in parasite proteins has also been described in chronic patients in the asymptomatic and cardiac phases of the disease [19,45,46].

When GSEA analysis was applied to the expression data of CCC I patients, it was observed that the same gene sets which were upregulated in patients with indeterminate form of Chagas disease compared to healthy donors [15] were also enriched in patients with cardiac symptomatology with respect to HD. These gene sets were involved in “antigen-dependent B cell activation”, “stress induction of HSP regulation”, “cytokines and inflammatory response” and “NO2-dependent IL12 pathway in NK cells”, each of which may play a critical role in the response to infection [15]. This enrichment is explained by the upregulation of certain genes that are correlated with PC1 (the principal component that explains the differences between CCC I and HD subjects) and/or showing a statistically significant differential gene expression in the volcano plot (*p* < 0.05; FC > 0.6). In addition, the “IL10 anti-inflammatory signaling pathway” was also enriched in CCC I patients. IL10 is a cytokine produced by T-helper 2 cells, B cells and activated macrophages [47], and is known to play a major role in controlling both innate and adaptive immunity. Its primary function is to limit and ultimately terminate inflammatory responses. IL10 has shown to protect the host in various parasitic diseases, such as malaria, where this cytokine counteracts the proinflammatory response induced by *P*. *falciparum*, and where deficiency of this host protection mechanism may occur in severe forms [48]. IL10 downregulates macrophages to become more anti-inflammatory type, thus may maintain the balance between pathogenic and protective immune responses [49]. Other studies have described that IL10 also downregulates the production of other cytokines such as IFNγ and TNFα through classically activated macrophages (M1) [50, 51]. In addition, polymorphisms affecting *IL12B* and *IL10* genes have recently been described as playing a role in the genetic susceptibility to the development of chagasic cardiomyopathy [52] and that low *IL10* expression was associated with worse cardiac function [53]. The fact that CCC I patients, who do not have severe cardiac disease, have this IL10 pathway enriched could indicate the important involvement of regulatory mechanisms to limit generation of excessive inflammatory responses, hence they can avoid the cardiac damage that is associated with the maintenance of inflammation by the persistence of the parasite [16,54–56].

Interestedly, important differences in particularly 9 differentially expressed genes were identified between CCC I and IND. All DEGs were upregulated in CCC I patients compared to IND patients with the exception of *GZMM* which was downregulated. It has been reported that granzyme M mediates a unique cell death pathway characterized by swelling of a target cell and vacuolisation [57] and also that it may be important for the cytotoxic potential of memory and effector CD8^+^ T cells [58]. Thus, the downregulation of *GZMM* observed in CCC I patients could be related to a lower cytotoxic capacity and a worse control of the infection leading to the progression of IND patients towards more severe forms of the disease. The higher expression of the *IFNG*, *IL1B*, *IL5*, *IL5RA* and *IL6* genes varied with statistical significance in IND *versus* HD, such as CCC I *versus* HD. This result indicates that although these are genes whose expression is increased in response to *T*. *cruzi* infection, their expression is exacerbated in patients with cardiac symptomatology. The remaining upregulated genes in CCC I *versus* IND were *CCR5*, the immunomodulatory cytokine *IL10* and its receptor *IL10RB*. These results are in agreement with previous studies of other authors that reported a higher expression of genes encoding the inflammatory cytokines IFN-γ, IL-6, and IL-1β in CCC than in IND patients [59]. In addition, other authors have associated an increased production of IL6 with a higher percentage of inflammatory monocytes in these patients with cardiac Chagas disease [60]. On the other hand, activation of the proinflammatory cytokine IL1B has been proposed as one of the mechanisms that promote fibrosis and inflammation in the heart [61]. Thus, an increased level of *IL1B* expression has been observed in CCC patients, which has led to propose the different activation pathways of this cytokine as a therapeutic target to prevent the evolution of cardiomyopathy, fibrosis and inflammation in Chagas disease [62].

Among upregulated genes, *CCR5* was the one with the greatest difference in its expression level showing 6 times more expression in CCC I than in IND patients. Other authors have described that the Th1 immune response is exacerbated in CCC patients and that *CCR5* expression in PBMCs was higher in patients with cardiomyopathy compared to IND patients and uninfected subjects [63]. Increased mRNA expression of *CCR5*, as well as other chemokine receptors, has also been reported in the myocardium of patients with CCC [12, 40]. In addition, it was observed that *CCR5* and *CXCR4* expression was different on the surface of peripheral blood mononuclear cells from patients with chronic chagasic cardiomyopathy and healthy subjects [64]. Likewise, patients with mild CCC had leukocytes with elevated expression of *CCR5* compared to healthy subjects [62]. The differential expression of both receptors on leukocytes of patients with CCC was consistent and clearly correlated with the degree of heart function such that the lower the heart function, the lower the expression of *CCR5* or *CXCR4* [64]. The upregulation of *CCR5* observed in this study in patients with CCC I is in line with this observation and with the hypothesis of the possible involvement of the chemokine system in early forms of chagasic cardiomyopathy [64]. However, although CCR5 has been associated with the maintenance of inflammation and the pathogenesis of chagasic myocarditis [65,66], there is no consensus since other authors have involved CCR5 with the control of *T*. *cruzi* replication [65]. Regardless of whether it has a favorable or unfavorable role, increased *CCR5* expression could be an interesting candidate as a biomarker of heart disease progression in patients with Chagas disease that could help control and/or prevent the onset of more severe manifestations. Furthermore, the results of the present study revealed that an increase in *IFNG* expression occurred in patients with CCC I *versus* IND, as well as an upregulation of *IL10* gene. Thus, our results are consistent with other authors observations who have reported a higher number of IFN-γ-producing T cells in peripheral blood from CCC compared to IND [13] and an increased IL10 production in CCC patients in the milder form of the disease [67]. These data could suggest that patients in the Kuschnir I stage (an early cardiac stage following Kuschnir classification) still possess active regulatory T cells that may play a role in controlling the intensity of inflammation in chronic Chagas disease.

With the use of OpenArray technology, we have been able to evaluate the expression of a large collection of genes simultaneously and in parallel in several subjects with chronic Chagas disease and healthy subjects. Therefore, this study provides new insights into the pathogenesis of Chagas disease describing the existence of differential expression patterns in certain genes involved in the antigen-specific cellular immune response that takes place in patients with CCC I *versus* asymptomatic IND as well as *versus* healthy subjects. The identification of these genes, as well as the relevant immunological pathways in which they appear to be involved, may represent promising molecules to be evaluated as useful biomarkers of disease progression, particularly in the early cardiac phase of chronic patients (Kuschnir I). Furthermore, the identification of the set of these genes is very valuable as targets in the context of diagnosis, immunotherapy strategies such as vaccine development and the development of immunomodulatory compounds that would undoubtedly be very useful to prevent the progression to more severe CCC forms and the high morbidity and mortality rates associated to these forms of this neglected disease.

## Supporting information

S1 Fig Top-10 most stable genes according to GeNorm, NormFinder and RefFinder algorithms. Gene expression stability is represented as bars, with expression being more stable at lower M-value (GeNorm), stability value (NormFinder) or geomean of ranking values (RefFinder). The corresponding stability ranking of all analyzed genes is indicated. Classical reference genes are shown as grey bars.
(TIF)

S2 Fig Principal-component analysis (PCA) in individual and blended samples from cardiac Chagas disease patients. PCA was applied on NRQ (Normalized Relative Quantities) values of gene expression of 106 analyzed genes in (A) CCC I blended patients (yellow circles) and CCC I individual patients (black rhombus) and in (B) HD (blue circles), CCC I blended patients (yellow circles) and CCC I individual patients (black rhombus). PC1 and PC2 are plotted on the x and y axes, respectively, and the proportion of variance captured for both components is given as a percentage. The results were confirmed by a two-tailed unpaired t-test: (A) There were no statistically significant differences between the scores obtained in the two groups for each component (PC1 *p* = 0.06, PC2 *p* = 0.86, PC3 *p* = 0.90); (B) Statistically significant differences were observed both between HD and CCC I individual patients (*p* < 0.0001) and between HD and CCC I blended patients (*p* < 0.0001).
(TIF)

S3 Fig Principal component analysis (PCA) of gene expression profiles in cardiac and indeterminate Chagas disease patients. PCA was applied on NRQ values of 106 analyzed genes from cardiac Chagas disease patients (CCC I, orange triangles) and indeterminate patients (IND, black squares). (A) PCA score plot of Principal Components 1 (PC1) and 2 (PC2) on the y and x-axis, respectively. (B) PCA score plot of Principal Components 1 (PC1) and 4 (PC4) on the x and y-axis, respectively. The proportion of total variance related to each principal component is given as a percentage and indicated on the axis next to the corresponding principal component.
(TIF)

S4 Fig Heatmap of the top-100 genes determined by GSEA in indeterminate and cardiac Chagas disease patients. The values of the expression level of each gene are represented as colors, ranging from dark red to dark blue, based on the highest and lowest normalized relative quantity (NRQ) value of each gene, respectively. The genes represented in vertical order from the top to the bottom are: *ICOS*, *GATA3*, *CD28*, *GZMM*, *CD48*, *CD3E*, *TGFBR2*, *ICOSLG*, *SELL*, *BCL2*, *CD160*, *MKI67*, *IL18*, *KLRG1*, *CD2*, *NOS2*, *IL2RG*, *TNF*, *STAT1*, *GZMK*, *CD4*, *CSF2*, *ITGA4*, *CD27*, *ITGAL*, *CD40LG*, *CXCR3*, *LAG3*, *CTLA4*, *IL12A*, *IL17A*, *CCL5*, *IL10RA*, *CSF1*, *ITGB2*, *LGALS9*, *IL17RA*, *FCER1A*, *TBX21*, *TGFB1*, *BTLA*, *XCR1*, *CD8A*, *IL2*, *STAT3*, *CR2*, *IL7R*, *IL6R*, *IL12B*, *TNFSF10*, *CD83*, *ICAM1*, *IL4R*, *IL10RB*, *CD274*, *TNFRSF14*, *IL1B*, *IL10*, *IL2RA*, *HAVCR2*, *FCER2*, *CLEC9A*, *CCR5*, *IL7*, *IDO1*, *TGFB2*, *IFNGR2*, *GZMB*, *IL5RA*, *CD80*, *FNAR1*, *FASLG*, *CD69*, *TGFBR1*, *PRF1*, *PDCD1LG2*, *IFNG*, *CCR7*, *IL12RB1*, *IL6*, *IL12RB2*, *IFNGR1*, *CD19*, *NCAM1*, *ITGAX*, *IL23R*, *CASP3*, *IL13*, *GZMH*, *CCR1*, *CD86*, *PDCD1*, *IL18R1*, *CD40*, *B3GAT1*, *CD58*, *IL23A*, *IL27*, *FOXP3* and *IL5*.
(TIF)

S1 Table TaqMan assays used for RT-qPCR analysis using QuantStudio 12K Flex Real-Time PCR System (n = 112). (DOCX)

S2 Table PC1-correlated genes. Genes with factor loading of Principal Component 1 (PC1) higher than 0.6 or lower than -0.6 from the Principal Component Analysis (PCA) applied on the normalized relative quantities (NRQ) of cardiac Chagas disease patients and healthy donors.
(DOCX)
