# Supplementary figures and images for: Differential expression profile of genes involved in the immune response associated to progression of chronic Chagas disease
Source: PLoS Negl Trop Dis. 2023 Jul 13;17(7):e0011474. doi: 10.1371/journal.pntd.0011474 (PMC10368263; doi:10.1371/journal.pntd.0011474)

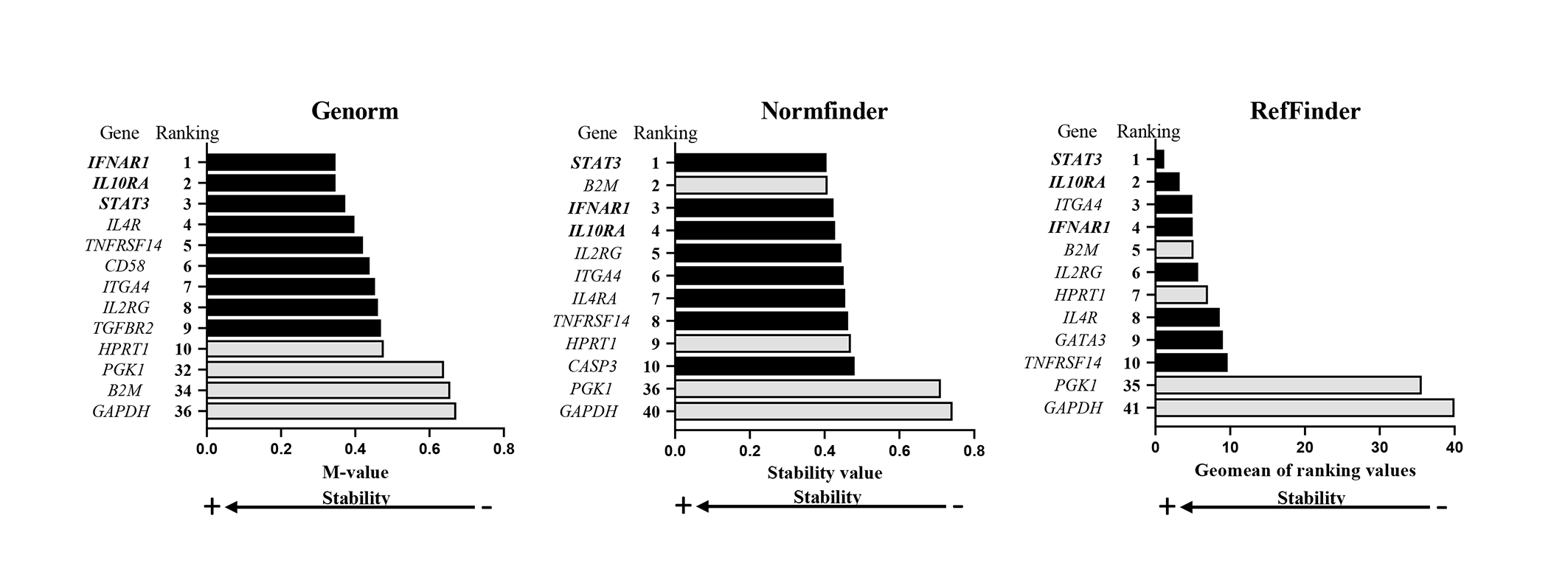

Supplement: S1 Fig — Gene expression stability is represented as bars, with expression being more stable at lower M-value (GeNorm), stability value (NormFinder) or geomean of ranking values (RefFinder). The corresponding stability ranking of all analyzed genes is indicated. Classical reference genes are shown as grey bars. (TIF) [file pntd.0011474.s001.tif]

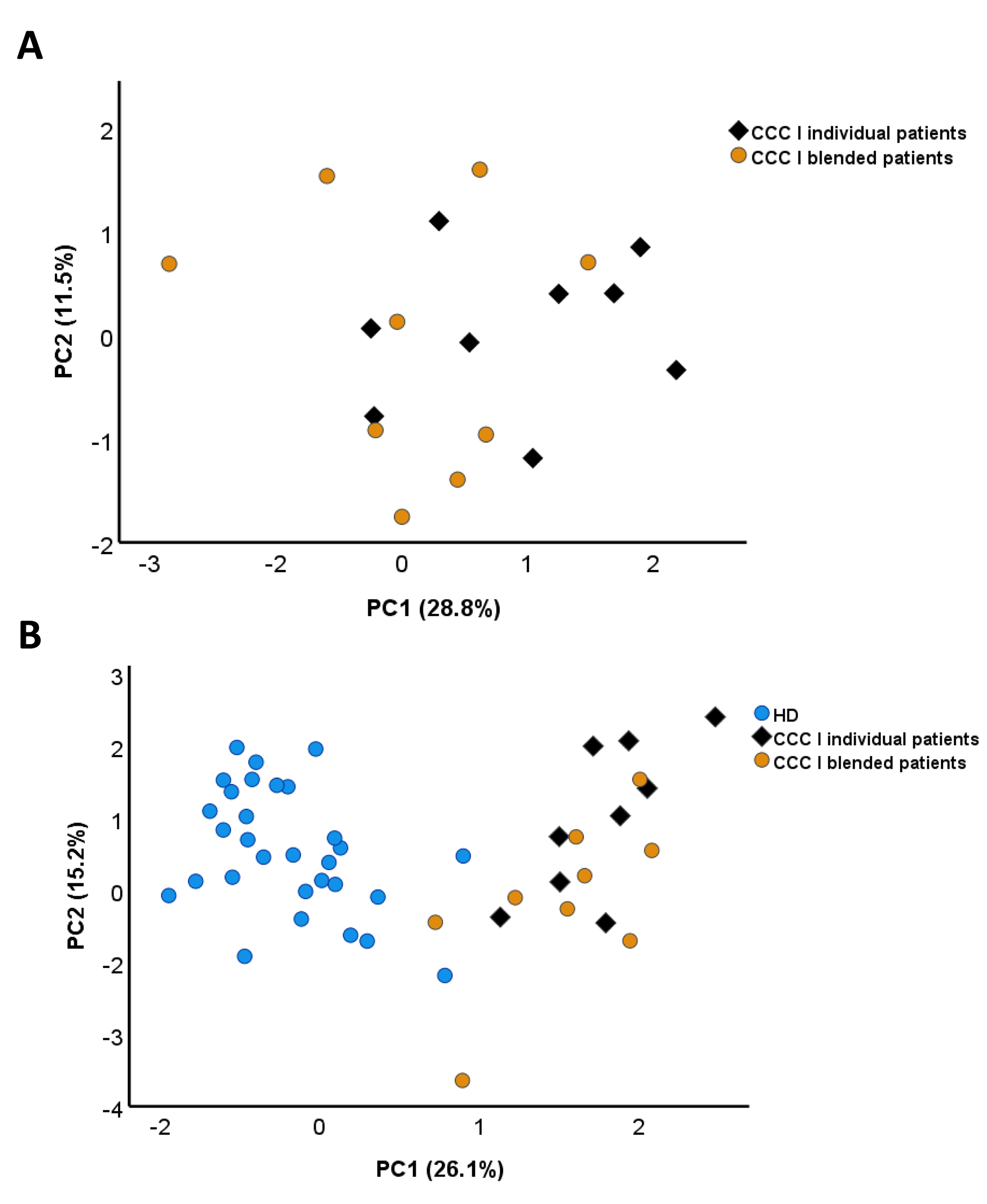

Supplement: S2 Fig — PCA was applied on NRQ (Normalized Relative Quantities) values of gene expression of 106 analyzed genes in (A) CCC I blended patients (yellow circles) and CCC I individual patients (black rhombus) and in (B) HD (blue circles), CCC I blended patients (yellow circles) and CCC I individual patients (black rhombus). PC1 and PC2 are plotted on the x and y axes, respectively, and the proportion of variance captured for both components is given as a percentage. The results were confirmed by a two-tailed unpaired t-test: (A) There were no statistically significant differences between the scores obtained in the two groups for each component (PC1 p = 0.06, PC2 p = 0.86, PC3 p = 0.90); (B) Statistically significant differences were observed both between HD and CCC I individual patients (p < 0.0001) and between HD and CCC I blended patients (p < 0.0001). (TIF) [file pntd.0011474.s002.tif]

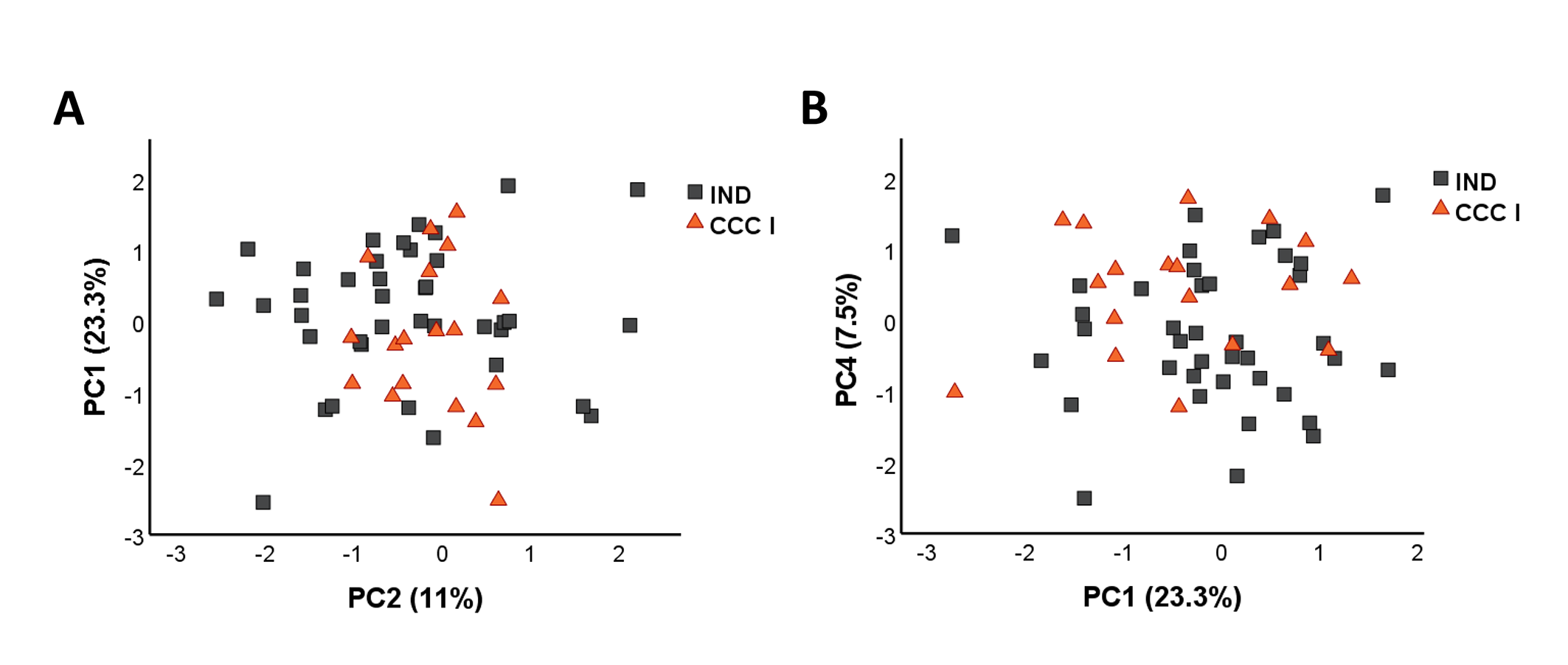

Supplement: S3 Fig — PCA was applied on NRQ values of 106 analyzed genes from cardiac Chagas disease patients (CCC I, orange triangles) and indeterminate patients (IND, black squares). (A) PCA score plot of Principal Components 1 (PC1) and 2 (PC2) on the y and x-axis, respectively. (B) PCA score plot of Principal Components 1 (PC1) and 4 (PC4) on the x and y-axis, respectively. The proportion of total variance related to each principal component is given as a percentage and indicated on the axis next to the corresponding principal component. (TIF) [file pntd.0011474.s003.tif]

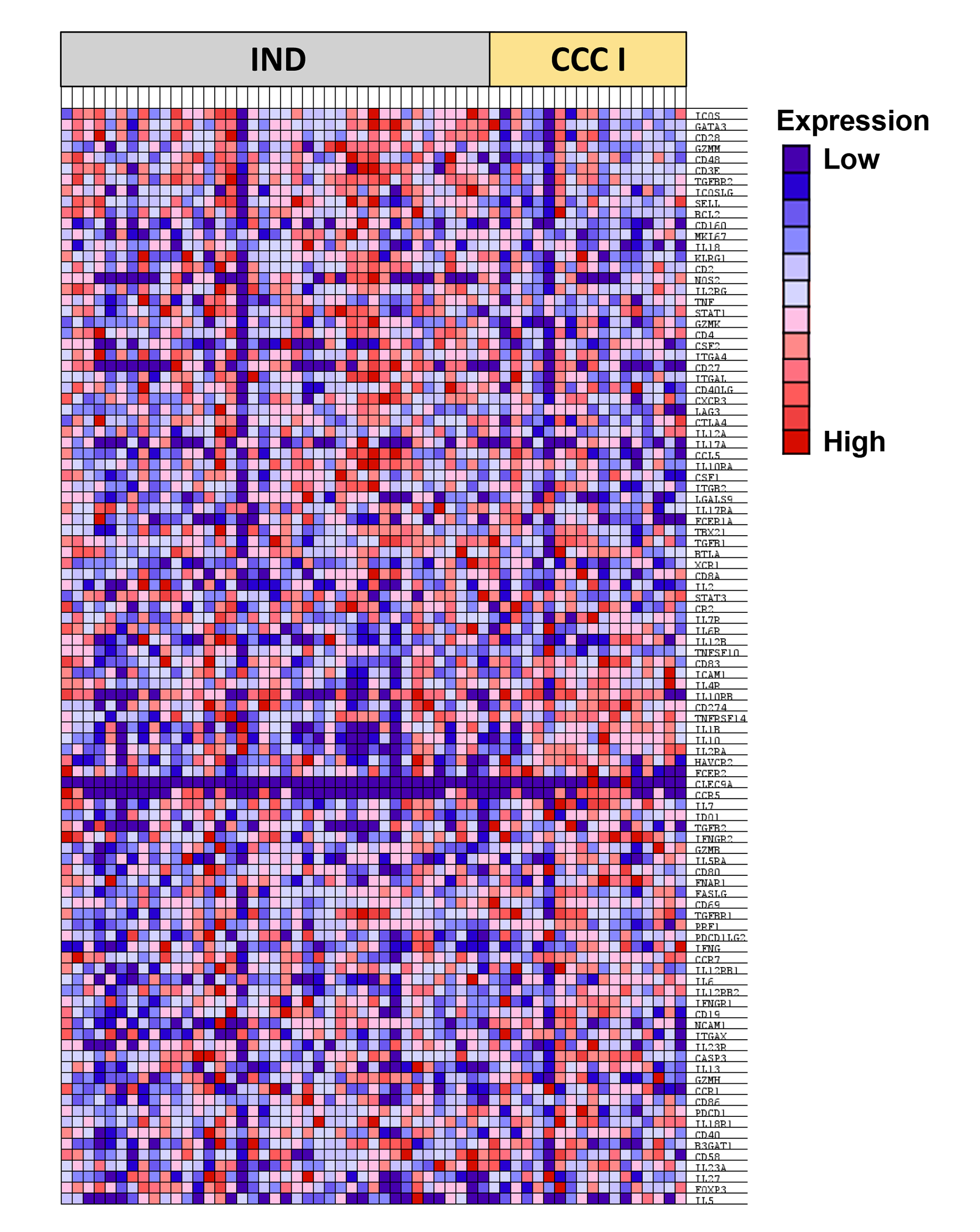

Supplement: S4 Fig — The values of the expression level of each gene are represented as colors, ranging from dark red to dark blue, based on the highest and lowest normalized relative quantity (NRQ) value of each gene, respectively. The genes represented in vertical order from the top to the bottom are: ICOS, GATA3, CD28, GZMM, CD48, CD3E, TGFBR2, ICOSLG, SELL, BCL2, CD160, MKI67, IL18, KLRG1, CD2, NOS2, IL2RG, TNF, STAT1, GZMK, CD4, CSF2, ITGA4, CD27, ITGAL, CD40LG, CXCR3, LAG3, CTLA4, IL12A, IL17A, CCL5, IL10RA, CSF1, ITGB2, LGALS9, IL17RA, FCER1A, TBX21, TGFB1, BTLA, XCR1, CD8A, IL2, STAT3, CR2, IL7R, IL6R, IL12B, TNFSF10, CD83, ICAM1, IL4R, IL10RB, CD274, TNFRSF14, IL1B, IL10, IL2RA, HAVCR2, FCER2, CLEC9A, CCR5, IL7, IDO1, TGFB2, IFNGR2, GZMB, IL5RA, CD80, FNAR1, FASLG, CD69, TGFBR1, PRF1, PDCD1LG2, IFNG, CCR7, IL12RB1, IL6, IL12RB2, IFNGR1, CD19, NCAM1, ITGAX, IL23R, CASP3, IL13, GZMH, CCR1, CD86, PDCD1, IL18R1, CD40, B3GAT1, CD58, IL23A, IL27, FOXP3 and IL5. (TIF) [file pntd.0011474.s004.tif]
