# Supplementary material for: Differential expression profile of genes involved in the immune response associated to progression of chronic Chagas disease
Source: PLoS Negl Trop Dis. 2023 Jul 13;17(7):e0011474. doi: 10.1371/journal.pntd.0011474 (PMC10368263; doi:10.1371/journal.pntd.0011474)
Supplement: S1 Table — (DOCX) [file pntd.0011474.s005.docx]

**S1 Table. TaqMan assays used for RT-qPCR analysis using QuantStudio™ 12K Flex Real-Time PCR System (n=112).**

| **Nº** | **Gene Symbol** | **Assay ID** | **Gene Name** |
| --- | --- | --- | --- |
| 1 | *ACTB* | Hs99999903_m1 | actin beta |
| 2 | *B2M* | Hs99999907_m1 | beta-2-microglobulin |
| 3 | B3GAT1 | Hs00218629_m1 | beta-1,3-glucuronyltransferase 1 |
| 4 | BCL2 | Hs00153350_m1 | BCL2, apoptosis regulator |
| 5 | BTLA | Hs00699198_m1 | B and T lymphocyte associated |
| 6 | CASP3 | Hs00234387_m1 | caspase 3 |
| 7 | CCL5 | Hs00982282_m1 | C-C motif chemokine ligand 5 |
| 8 | CCR1 | Hs00174298_m1 | C-C motif chemokine receptor 1 |
| 9 | CCR5 | Hs00152917_m1 | C-C motif chemokine receptor 5 (gene/pseudogene) |
| 10 | CCR7 | Hs01013469_m1 | C-C motif chemokine receptor 7 |
| 11 | CD160 | Hs00199894_m1 | CD160 molecule |
| 12 | CD19 | Hs00174333_m1 | CD19 molecule |
| 13 | **CD1A** | **Hs00233332_m1** | **CD1a molecule** |
| 14 | CD2 | Hs00233515_m1 | CD2 molecule |
| 15 | CD27 | Hs00386811_m1 | CD27 molecule |
| 16 | CD274 | Hs01125301_m1 | CD274 molecule |
| 17 | CD28 | Hs01007422_m1 | CD28 molecule |
| 18 | CD3E | Hs01062241_m1 | CD3e molecule |
| 19 | CD4 | Hs01058407_m1 | CD4 molecule |
| 20 | CD40 | Hs00374176_m1 | CD40 molecule |
| 21 | CD40LG | Hs00163934_m1 | CD40 ligand |
| 22 | CD48 | Hs00152927_m1 | CD48 molecule |
| 23 | CD58 | Hs00156385_m1 | CD58 molecule |
| 24 | CD69 | Hs00934033_m1 | CD69 molecule |
| 25 | CD80 | Hs00175478_m1 | CD80 molecule |
| 26 | CD83 | Hs00188486_m1 | CD83 molecule |
| 27 | CD86 | Hs01567026_m1 | CD86 molecule |
| 28 | CD8A | Hs00233520_m1 | CD8a molecule |
| 29 | CLEC9A | Hs01651638_m1 | C-type lectin domain family 9 member A |
| 30 | CR2 | Hs00153398_m1 | complement component 3d receptor 2 |
| 31 | CSF1 | Hs00174164_m1 | colony stimulating factor 1 |
| 32 | CSF2 | Hs00929873_m1 | colony stimulating factor 2 |
| 33 | CTLA4 | Hs00175480_m1 | cytotoxic T-lymphocyte associated protein 4 |
| 34 | CXCR3 | Hs00171041_m1 | C-X-C motif chemokine receptor 3 |
| 35 | FAS | Hs00236330_m1 | Fas cell surface death receptor |
| 36 | FASLG | Hs00181225_m1 | Fas ligand |
| 37 | FCER1A | Hs00758599_m1 | Fc fragment of IgE receptor Ia |
| 38 | FCER2 | Hs00233627_m1 | Fc fragment of IgE receptor II |
| 39 | FOXP3 | Hs01085834_m1 | forkhead box P3 |
| 40 | *GAPDH* | Hs99999905_m1 | glyceraldehyde-3-phosphate dehydrogenase |
| 41 | GATA3 | Hs00231122_m1 | GATA binding protein 3 |
| 42 | GZMA | Hs00196206_m1 | granzyme A |
| 43 | GZMB | Hs00188051_m1 | granzyme B |
| 44 | GZMH | Hs00277212_m1 | granzyme H |
| 45 | GZMK | Hs00157878_m1 | granzyme K |
| 46 | GZMM | Hs00193417_m1 | granzyme M |
| 47 | HAVCR2 | Hs00262170_m1 | hepatitis A virus cellular receptor 2 |
| 48 | *HPRT1* | Hs99999909_m1 | hypoxanthine phosphoribosyltransferase 1 |
| 49 | ICAM1 | Hs00164932_m1 | intercellular adhesion molecule 1 |
| 50 | ICOS | Hs00359999_m1 | inducible T-cell costimulator |
| 51 | ICOSLG | Hs00323621_m1 | inducible T-cell costimulator ligand |
| 52 | IDO1 | Hs00984148_m1 | indoleamine 2,3-dioxygenase 1 |
| 53 | IFNAR1 | Hs01066116_m1 | interferon alpha and beta receptor subunit 1 |
| 54 | IFNG | Hs00989291_m1 | interferon gamma |
| 55 | IFNGR1 | Hs00988304_m1 | interferon gamma receptor 1 |
| 56 | IFNGR2 | Hs00194264_m1 | interferon gamma receptor 2 (interferon gamma transducer 1) |
| 57 | IL10 | Hs00961622_m1 | interleukin 10 |
| 58 | IL10RA | Hs00155485_m1 | interleukin 10 receptor subunit alpha |
| 59 | IL10RB | Hs00175123_m1 | interleukin 10 receptor subunit beta |
| 60 | IL12A | Hs00168405_m1 | interleukin 12A |
| 61 | IL12B | Hs00233688_m1 | interleukin 12B |
| 62 | IL12RB1 | Hs00234651_m1 | interleukin 12 receptor subunit beta 1 |
| 63 | IL12RB2 | Hs00155486_m1 | interleukin 12 receptor subunit beta 2 |
| 64 | IL13 | Hs00174379_m1 | interleukin 13 |
| 65 | IL17A | Hs00174383_m1 | interleukin 17A |
| 66 | IL17RA | Hs01064648_m1 | interleukin 17 receptor A |
| 67 | IL18 | Hs01038788_m1 | interleukin 18 |
| 68 | IL18R1 | Hs00175381_m1 | interleukin 18 receptor 1 |
| 69 | IL1B | Hs01555410_m1 | interleukin 1 beta |
| 70 | IL2 | Hs00174114_m1 | interleukin 2 |
| 71 | IL23A | Hs00372324_m1 | interleukin 23 subunit alpha |
| 72 | IL23R | Hs00332759_m1 | interleukin 23 receptor |
| 73 | **IL25** | **Hs03044841_m1** | **interleukin 25** |
| 74 | IL27 | Hs00377366_m1 | interleukin 27 |
| 75 | IL2RA | Hs00166229_m1 | interleukin 2 receptor subunit alpha |
| 76 | IL2RG | Hs00953624_m1 | interleukin 2 receptor subunit gamma |
| 77 | IL4 | Hs00174122_m1 | interleukin 4 |
| 78 | IL4R | Hs00965056_m1 | interleukin 4 receptor |
| 79 | IL5 | Hs00174200_m1 | interleukin 5 |
| 80 | IL5RA | Hs00602482_m1 | interleukin 5 receptor subunit alpha |
| 81 | IL6 | Hs00985639_m1 | interleukin 6 |
| 82 | IL6R | Hs01075666_m1 | interleukin 6 receptor |
| 83 | IL7 | Hs00174202_m1 | interleukin 7 |
| 84 | IL7R | Hs00233682_m1 | interleukin 7 receptor |
| 85 | ITGA4 | Hs00168433_m1 | integrin subunit alpha 4 |
| 86 | ITGAL | Hs00158218_m1 | integrin subunit alpha L |
| 87 | ITGAX | Hs00174217_m1 | integrin subunit alpha X |
| 88 | ITGB2 | Hs00164957_m1 | integrin subunit beta 2 |
| 89 | KLRG1 | Hs00195153_m1 | killer cell lectin like receptor G1 |
| 90 | LAG3 | Hs00158563_m1 | lymphocyte activating 3 |
| 91 | LGALS9 | Hs00247135_m1 | galectin 9 |
| 92 | MKI67 | Hs01032443_m1 | marker of proliferation Ki-67 |
| 93 | NCAM1 | Hs00941830_m1 | neural cell adhesion molecule 1 |
| 94 | NOS2 | Hs01075529_m1 | nitric oxide synthase 2 |
| 95 | PDCD1 | Hs01550088_m1 | programmed cell death 1 |
| 96 | PDCD1LG2 | Hs01057777_m1 | programmed cell death 1 ligand 2 |
| 97 | *PGK1* | Hs99999906_m1 | phosphoglycerate kinase 1 |
| 98 | PRF1 | Hs00169473_m1 | perforin 1 |
| 99 | SELL | Hs01046459_m1 | selectin L |
| 100 | STAT1 | Hs01013996_m1 | signal transducer and activator of transcription 1 |
| 101 | STAT3 | Hs00374280_m1 | signal transducer and activator of transcription 3 |
| 102 | *TBP* | Hs99999910_m1 | TATA-box binding protein |
| 103 | TBX21 | Hs00203436_m1 | T-box 21 |
| 104 | TGFB1 | Hs00998133_m1 | transforming growth factor beta 1 |
| 105 | TGFB2 | Hs00234244_m1 | transforming growth factor beta 2 |
| 106 | TGFBR1 | Hs00610320_m1 | transforming growth factor beta receptor 1 |
| 107 | TGFBR2 | Hs00234253_m1 | transforming growth factor beta receptor 2 |
| 108 | TNF | Hs00174128_m1 | tumor necrosis factor |
| 109 | TNFRSF14 | Hs00998604_m1 | TNF receptor superfamily member 14 |
| 110 | TNFRSF1A | Hs00533560_m1 | TNF receptor superfamily member 1A |
| 111 | TNFSF10 | Hs00921974_m1 | tumor necrosis factor superfamily member 10 |
| 112 | XCR1 | Hs00245540_s1 | X-C motif chemokine receptor 1 |

Classical reference genes according to literature are indicated in italics. Bold letters indicate genes with missing qPCR data*.*
