# Supplementary material for: Differential expression profile of genes involved in the immune response associated to progression of chronic Chagas disease
Source: PLoS Negl Trop Dis. 2023 Jul 13;17(7):e0011474. doi: 10.1371/journal.pntd.0011474 (PMC10368263; doi:10.1371/journal.pntd.0011474)
Supplement: S2 Table — Genes with factor loading of Principal Component 1 (PC1) higher than 0.6 or lower than -0.6 from the Principal Component Analysis (PCA) applied on the normalized relative quantities (NRQ) of cardiac Chagas disease patients and healthy donors. (DOCX) [file pntd.0011474.s006.docx]

**S2 Table. PC1-correlated genes**. Genes with factor loading of Principal Component 1 (PC1) higher than 0.6 or lower than -0.6 from the Principal Component Analysis (PCA) applied on the normalized relative quantities (NRQ) of cardiac Chagas disease patients and healthy donors.

| **Gene** | **Factor Loading for PC1** |
| --- | --- |
| ***FAS*** | 0.911 |
| ***BTLA*** | 0.807 |
| ***CSF2*** | 0.781 |
| ***CASP3*** | 0.78 |
| ***CD274*** | 0.78 |
| ***IL12A*** | 0.772 |
| ***IL12RB1*** | 0.77 |
| ***IL7*** | 0.769 |
| ***CD83*** | 0.766 |
| ***IL12RB2*** | 0.765 |
| ***CD40*** | 0.727 |
| ***IL6*** | 0.723 |
| ***IL2RA*** | 0.716 |
| ***TBX21*** | 0.711 |
| ***IL23A*** | 0.71 |
| ***STAT1*** | 0.695 |
| ***CSF1*** | 0.69 |
| ***IFNG*** | 0.684 |
| ***BCL2*** | 0.682 |
| ***CD40LG*** | 0.678 |
| ***CD69*** | 0.665 |
| ***TNF*** | 0.648 |
| ***IL5RA*** | 0.646 |
| ***CD80*** | 0.618 |
| ***IL2RG*** | 0.612 |
| ***CD2*** | 0.6 |
| ***TGFBR1*** | -0.607 |
| ***CCR1*** | -0.627 |
| ***ITGB2*** | -0.7 |
| ***IL18*** | -0.712 |
| ***IFNGR2*** | -0.736 |
| ***IFNGR1*** | -0.754 |
| ***CD86*** | -0.757 |
| ***ITGAX*** | -0.759 |
| ***HAVCR2*** | -0.765 |
| ***IL17RA*** | -0.79 |
